# Radial spoke protein 9 is necessary for axoneme assembly in *Plasmodium* but not in trypanosomatid parasites

**DOI:** 10.1242/jcs.260655

**Published:** 2023-06-08

**Authors:** Chandra Ramakrishnan, Cécile Fort, Sara Rute Marques, David J. P. Ferguson, Marion Gransagne, Jake Baum, Soraya Chaouch, Elisabeth Mouray, Linda Kohl, Richard J. Wheeler, Robert E. Sinden

**Affiliations:** ^1^ Department of Life Sciences, Imperial College London, London SW7 2AZ, UK; ^2^ Peter Medawar Building for Pathogen Research, University of Oxford, Oxford OX1 3SY, UK; ^3^ Nuffield Department of Clinical Laboratory Science, University of Oxford, Oxford OX3 9DU, UK; ^4^ Department of Biological and Medical Sciences, Oxford Brookes University, Oxford OX3 0BP, UK; ^5^ UMR 7245 Molécules de Communication et Adaptation des Micro-organismes, Muséum National d'Histoire Naturelle, Centre National de la Recherche Scientifique (CNRS), CP52, 61 rue Buffon, 75231 Paris Cedex 05, France

**Keywords:** Radial spoke, Flagella, Motility, Trypanosomatids, *Plasmodium*

## Abstract

Flagella are important for eukaryote cell motility, including in sperm, and are vital for life cycle progression of many unicellular eukaryotic pathogens. The ‘9+2’ axoneme in most motile flagella comprises nine outer doublet and two central-pair singlet microtubules. T-shaped radial spokes protrude from the outer doublets towards the central pair and are necessary for effective beating. We asked whether there were radial spoke adaptations associated with parasite lineage-specific properties in apicomplexans and trypanosomatids. Following an orthologue search for experimentally uncharacterised radial spoke proteins (RSPs), we identified and analysed RSP9. *Trypanosoma brucei* and *Leishmania mexicana* have an extensive RSP complement, including two divergent RSP9 orthologues, necessary for flagellar beating and swimming. Detailed structural analysis showed that neither orthologue is needed for axoneme assembly in *Leishmania*. In contrast, *Plasmodium* has a reduced set of RSPs including a single RSP9 orthologue, deletion of which in *Plasmodium berghei* leads to failure of axoneme formation, failed male gamete release, greatly reduced fertilisation and inefficient life cycle progression in the mosquito. This indicates contrasting selection pressures on axoneme complexity, likely linked to the different mode of assembly of trypanosomatid versus *Plasmodium* flagella.

## Teaser

**Summary:** Radial spoke head protein 9 (RSP9) is found in the flagella of trypanosomatids and malaria parasites, and is important for motility in trypanosomatids but critical for fertilisation in *Plasmodium*.

## INTRODUCTION

Motile eukaryotic cilia and flagella fulfil essential roles in cell movement or moving extracellular material past a cell (Wallmeier et al., 2020). The core of cilia and flagella is the axoneme, a cylindrical structure composed of nine outer microtubule doublets. Motile cilia (also called flagella) normally have two central single microtubules – the central pair, resulting in an overall ‘9+2’ axoneme organisation (Ishikawa, 2017; Mitchell and Smith, 2009). Radial spokes (RSs) assemble on the peripheral microtubule doublets and project towards to the central pair complex, which is composed of the central pair and associated proteins (Gui et al., 2021; Pigino and Ishikawa, 2012; Teves et al., 2016).

For successful generation of a flagellar beat, various structures are required in addition to the inner and outer dynein arms, which drive sliding between neighbouring doublet microtubules and thus flagellar bending (Petriman and Lorentzen, 2020). Such structures include the RSs. It is not known precisely how RSs are important for beating; however, a variety of evidence points either to direct mechanical roles or to mechanosensitive regulation of dynein activity through interaction with the central pair complex (Grossman-Haham et al., 2021; Oda et al., 2014; Viswanadha et al., 2017; Zhu et al., 2017). RSs are mushroom-shaped complexes (Hopkins, 1970; Chasey, 1974; Goodenough and Heuser, 1985) with the stalk attached to a microtubule doublet and the enlarged ‘head’ projecting towards the central pair of the axoneme. They are particularly well characterised in *Chlamydomonas reinhardtii*, in which mutagenesis has identified many mutants with defective RS assembly (Barber et al., 2012; Brokaw and Luck, 1985; Frey et al., 1997; Brokaw et al., 1982). RS molecular composition was elucidated using 2D gel electrophoresis (Piperno et al., 1981) and mass spectrometry, and, in *Chlamydomonas*, the RS is composed of at least 23 proteins (Yang et al., 2006). More recently, RS structure has been determined at atomic resolution by cryoelectron microscopy (Barber et al., 2012; Poghosyan et al., 2020).

It has been shown in *C. reinhardtii* that axonemes form by extension from the basal body, with nine peripheral microtubule doublets being extended. The nucleation of the central pair microtubules follows distal from the basal body in an area called the transition zone. The RSs are partially assembled in the cytoplasm to form the 12S complex (Qin et al., 2004). This complex is shaped like a ‘7’ and consists of radial spoke protein (RSP)1–RSP6, RSP7, RSP9 and RSP10–RSP12 (Diener et al., 2011). It is then transported by intraflagellar transport (IFT) and attached to the axoneme. It is hypothesised that the mature 20S spoke (Yang et al., 2001) is a dimer of the 12S complex (Diener et al., 2011).

Motile flagella are vital for many unicellular parasites (Ginger et al., 2008; Krüger and Engstler, 2015). Here, we wanted to study RSs in organisms with life cycles that are critically dependent on motile flagella, while displaying significant differences in assembly and lifespan of flagella. Our objective was to gain insight into RS adaptations that may be linked with parasitism-associated lineage-specific specialisations in flagellum biology. We selected two trypanosomatid species, *Trypanosoma brucei* and *Leishmania mexicana*, the agents of sleeping sickness and leishmaniasis, respectively, and *Plasmodium berghei*, responsible for rodent malaria and related to human malaria parasites.

In *T. brucei*, all life cycle stages have a motile flagellum, which is essential for motility, cell morphogenesis and progression through the insect vector, and pathogenesis in the mammalian host (Absalon et al., 2008; Fort et al., 2016; Kohl et al., 2003; Rotureau et al., 2014b; Shimogawa et al., 2018). In contrast, *Leishmania* does not require a long flagellum for cell division (Sunter et al., 2018), and the intracellular amastigote life cycle stage in the vertebrate host has a short 9+0 flagellum (Wheeler et al., 2015). Flagellar motility is, however, known to be necessary to infect the sandfly vector (Beneke et al., 2019), and the infectious life cycle stage transferred during a sandfly blood meal (the metacyclic stage) is highly motile (Findlay et al., 2021). Flagellum assembly in trypanosomatids requires a canonical IFT mechanism because flagellar formation occurs by axoneme growth, leading to progressive protrusion from the cell (Absalon et al., 2008; Fort et al., 2016; Gadelha et al., 2013; Sunter et al., 2018). For flagellar assembly, proteins are produced in the cytoplasm and loaded onto IFT particles near the base of the flagellum. IFT particles enter the flagellum in linear arrays called ‘trains’ that move along the microtubules using motor proteins powered by ATP: kinesins transport the IFT particles to the distal tip, where the cargo is released and the IFT particles are reorganised. Dyneins are responsible for return traffic to the flagellum base. Growing flagella gradually extend from the cell over the course of several hours in both *Trypanosoma* and *Leishmania* (Bastin et al., 1999; Sherwin et al., 1989; Kohl et al., 2003; Sunter et al., 2018; Wheeler et al., 2011).

In contrast, *Plasmodium* species have a flagellum only in one critical stage of the life cycle: during sexual development, when flagellate male gametes fertilise female gametes. This happens in the lumen of the mosquito midgut, which becomes infected after ingesting a blood meal containing mixed blood stages of *Plasmodium*, including gametocytes (intraerythrocytic male and female sexual stages that are quiescent in the vertebrate host). It is only in the insect midgut that the gametocytes are activated, and within 10 min they can form mature gametes (Sinden et al., 1976): the female gametes egress by rupture of the host cell (Andreadaki et al., 2018) with the help of proteins secreted from osmiophilic bodies (Ishino et al., 2020). Male gametocytes also escape from the ruptured erythrocyte but undergo a complex series of transformations, including three rounds of coordinated endomitosis and axoneme formation. The single microtubule organising centre (MTOC) in the activated microgametocyte produces eight basal bodies with nine single A microtubules, which are redistributed as the spindle poles migrate at each of the three mitotic divisions. Each basal body nucleates an axoneme in the cytoplasm (Sinden et al., 1976). In contrast to most known flagellate organisms and especially the related coccidians such as *Toxoplasma* gondii or *Eimeria*, *Plasmodium* lacks IFT (Avidor-Reiss et al., 2004; Briggs et al., 2004) and instead assembles the axonemes within the cytoplasm (Sinden et al., 1976) in an IFT-independent manner (Briggs et al., 2004). The cytoplasmic basal bodies are attached to the intranuclear mitotic spindle poles, and thus each is structurally linked to one haploid set of chromosomes (Sinden et al., 1978). After all the three mitotic divisions have taken place and the axonemes have formed, as each basal body is driven by the axoneme out of the cell, it draws the linked haploid genome into the emerging microgamete. The immotile axonemes initially lie parallel to the microgametocyte membrane, then on becoming motile, re-orient perpendicularly to the microgametocyte membrane and ‘swim’ emerging basal body foremost, simultaneously becoming enveloped by the plasmalemma to form the free microgametes (Sinden et al., 1976). Microgamete escape (termed ‘exflagellation’) is rapid, taking only seconds to minutes (Sinden and Croll, 1975). The microgamete (unlike many organisms including Coccidia) does not contain a mitochondrion and thus has only limited motility. The male gametes contact, and then fuse with, the female gamete to form a zygote; therein the axonemes become sessile (usually within 45 min of their original assembly), continuing the life cycle in the mosquito. Although they are built using different mechanisms, flagella from trypanosomatids and *Plasmodium* both display the canonical 9+2 microtubule architecture, with the axoneme microtubules nucleated at the basal body (Sinden et al., 1976, 2010; Sinden and Croll, 1975; Marques et al., 2015; Vaughan and Gull, 2016).

As there may be differences in RSs associated with these differing mechanisms of flagellum assembly, we aimed to study RS proteins that are essential for flagellar beating in these parasites. To identify RSPs of potential interest, we performed a comprehensive bioinformatic analysis of RSPs across eukaryotes. We found that, similar to the reference organism (*Chlamydomonas*), many organisms, including trypanosomatids, possess a large number of RSPs. In contrast, only three RSPs could be identified in *Plasmodium*: RSP3, RSP9 and RSP4/6 (Talman et al., 2014). We identified a weakly RSP7/11-like protein as non-flagellar and thus excluded it from comparative analysis. Of these, RSP3 has been studied previously in *T. brucei* (Ralston et al., 2006) and RSP4/6 in *T. brucei* and in *Leishmania* (Beneke et al., 2019; Gorilak et al., 2021; Wheeler et al., 2015). We therefore carried out our comparative analysis on the RS protein RSP9, which forms part of the enlarged ‘head’ of the RSP mushroom shape.

We determined that *T. brucei* and *L. mexicana* have two RSP9-like paralogues, reflecting innovation in RS architecture with a possibility of a heterodimeric protein structure. Using gene deletion in *L. mexicana*, we show that both RSP9 and RSP9-like are necessary for RS head assembly and normal cell motility but are not needed for cell survival. Similar results were obtained in the *TbRSP9L^RNAi^* cell line. The limited knockdown of RSP9 expression in the *TbRSP9^RNAi^* cell line, while causing a reduction in motility, could possibly explain why we could not detect loss of RS head electron density in this cell line. In contrast, loss of the single *rsp9* gene in *Plasmodium* causes a dramatic failure in axoneme assembly, disrupting male gamete exflagellation and impairing life cycle progression. We discuss the implications given the differing axoneme assembly mechanisms in the context of evolution of RS structure complexity and parasitism-associated streamlining.

## RESULTS

### Conservation of RS proteins

**Fig. 1.**
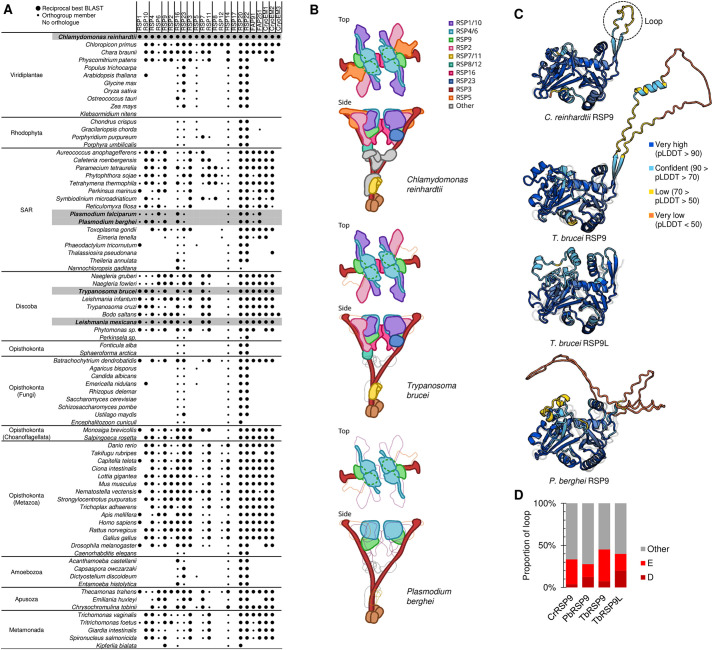
**Radial spoke proteins in eukaryotes.** (A) Presence of orthologues of *C. reinhardtii* radial spoke proteins (RSPs) in diverse flagellated and aflagellated eukaryotes, detected by either reciprocal best BLAST protein sequence search or presence of an orthogroup member. Matrix summarising the presence of at least one orthogroup member detected using OrthoFinder (small circle), a reciprocal best BLAST orthologue (large circle), or no (space) detectable orthologue of the RSP gene family. (B) Cartoon showing the structure of a *C. reinhardtii* radial spoke (RS) (top), and cartoons showing RSPs with (solid colour) or without (thin grey outlines) orthologues in *T. brucei* and *P. berghei* mapped on the *C. reinhardtii* RS structure (middle and bottom, respectively). (C) Predicted 3D structures of TbRSP9, TbRSP9L and PbRSP9 using AlphaFold compared to the experimentally determined *C. reinhardtii* RSP9 structure. Colours represent the predicted local distance difference test score (pLDDT) of the AlphaFold prediction, each shown aligned to the experimentally determined structure shown as a grey ghost. (D) Proportion of acidic amino acids (D, aspartic acid; E, glutamic acid) in the RSP9 loops. Loop length 27, 105, 82 and 5 amino acids for CrRSP9, PbRSP9, TbRSP9 and TbRSP9L, respectively.

To achieve a comprehensive analysis of highly conserved RS machinery, we first carried out a comprehensive bioinformatic analysis to identify orthologues of *C. reinhardtii* RSPs. We gathered a set of high-confidence *C. reinhardtii* RSPs using evidence from 2D gel electrophoresis and mass spectrometry (Luck et al., 1977; Diener et al., 2011; Piperno et al., 1977; Yang et al., 2006) and RS cryoelectron microscopy (Gui et al., 2021). These orthologues were RSP1–RSP23, with the exception of RSP13, RSP14, RSP18 and RSP21, for which the molecular identification remains unknown, along with flagellar-associated proteins FAP91 and FAP251 and three uncharacterised proteins [identified from the cryoelectron microscopy structure (Gui et al., 2021)] (Fig. 1A). RSP19 was excluded because it is β-tubulin.

To identify orthologues of these RSP proteins in *T. brucei*, *L. mexicana*, *Plasmodium falciparum* and *P. berghei*, we first used a reciprocal best protein BLAST search. Mindful that RSP1 and RSP10, RSP4 and RSP6, and RSP7 and RSP11, respectively, appear to be ancient paralogous pairs (which we refer to as the RSP1/10, RSP4/6 and RSP7/11 families), we then used Orthofinder (Emms and Kelly, 2015, 2019) to identify orthogroups in a diverse set of flagellate and aflagellate eukaryotes (Fig. 1A). Orthologues of some RSPs (RSP16, RSP23, RSP5 and RSP14) are found in many aflagellate species; thus, proteins in these orthogroups are also likely to have non-RSP functions. One such example is the orthologue of RSP16 as it contains a DNAJ protein domain (Pfam: PF00226), a common and abundant protein domain in most species. However, the only flagellum-localising DNAJ domain-containing protein in *T. brucei* was Tb927.11.15130 (Dean et al., 2017), which we therefore list as an RSP16 orthologue. Overall, this motivated our focus on orthologues of RSP1/10, RSP4/6 and RSP7/11 families along with RSP9, RSP2 and RSP3, which are almost exclusively found in flagellate species and are present in almost all flagellates. These proteins are most likely to have highly evolutionarily conserved RS functions.

Intriguingly, *T. brucei* and *L. mexicana* possess two RSP9 proteins: TbRSP9 (Tb927.8.810) and TbRSP9L (Tb927.11.2540), and LmRSP9 (LmxM.07.0930) and LmRSP9L (LmxM.32.2500), respectively. TbRSP9 is more similar to LmxRSP9 (55% identity) than to the TbRSP9L paralogue (25% identity), and phylogenetic analysis points to an ancient duplication in the kinetoplastid lineage (Fig. S1). Finally, we used TrypTag, the genome-wide protein localisation resource in *T. brucei* (Dean et al., 2017), to confirm that the identified *T. brucei* RSP orthologues (including both RSP9-like proteins) localised to the flagellum (Fig. S2).

These comprehensive analyses show that *T. brucei* and *L. mexicana* have orthologues of most of the RSPs identified to date (Fig. 1A; Table S1), while *Plasmodium* possesses only a single orthologue each of RSP3 (PBANKA_1039000), RSP9 (PBANKA_1431500) and RSP4/6 (PBANKA_0942300), all of which have also been detected by proteomic analysis of male gametes in *Plasmodium* (Talman et al., 2014). Additionally, we identified a tentative, non-annotated RSP7/11 family protein in *Plasmodium* (PBANKA_112580). However, upon analysis, the encoding gene is expressed in all *P. berghei* stages, and gene deletion does not abolish exflagellation or axoneme formation completely (Fig. S3), but results in a mild reduction of ookinete formation and, subsequently, similar numbers of oocysts compared to wild type (WT) and successful transmission, indicating that PBANKA_112580 lacks a vital flagellar function (Fig. S4) and is therefore unlikely to be an RSP.

These bioinformatic analyses highlight RSP9 as being well conserved but with parasite lineage-specific adaptation. The function of either predicted RS head protein (RSP9 or RSP4/6) has been analysed in *Plasmodium*, and RSP9 has a previously unrecognised duplication in *Trypanosoma* and *Leishmania*, neither of which has been experimentally analysed*.*

### RSP9s are flagellar proteins in *Trypanosoma*, *Leishmania* and *Plasmodium*

To illustrate where these RSP9 proteins may be found in the RS, we compared the predicted RS structures of *T. brucei* and *P. berghei* to the experimentally determined *C. reinhardtii* RS1 structure [PDB: 7JTK (Gui et al., 2021)] (Fig. 1B). This shows the dramatic difference in complexity, in terms of conserved RSP components, between these two parasites. RSP3 is in the stalk of the protein, and both RSP4/6 and RSP9 are in the head of the RS, suggesting that the minimal components for RS function are RSP3 to position the RS head components near the central pair complex and the RSP4/6–RSP9 tetramer core of each half of the RS head. TbRSP9 and TbRSP9L could both be aligned to the same region, and thus could assemble into homodimers or heterodimers. Using AlphaFold, we then compared the predicted tertiary structure of TbRSP9, TbRSP9L and PbRSP9 to *C. reinhardtii* RSP9 (Jumper et al., 2021; Mirdita et al., 2022; Wheeler, 2021) (Fig. 1C). As TbRSP9/LmRSP9 and TbRSP9L/LmRSP9L are very similar, only TbRSP9 and TbRSP9L are illustrated. Alignment of predicted RSP9 structures to the *C. reinhardtii* RSP9 structure determined by cryoelectron microscopy [PDB: 7JTK (Gui et al., 2021)] showed that the predicted *C. reinhardtii* structure is accurate (Fig. 1C) and that the TbRSP9 and PbRSP9 predicted structures are very similar to that of *C. reinhardtii* (Fig. 1C)*.* However, both TbRSP9 and PbRSP9 are predicted to have a large acidic loop in the position of the small unstructured loop not resolved by *C. reinhardtii* RS cryoelectron microscopy (Fig. 1C). This acidic loop appears analogous to those in RSP4 and RSP6 and sits on the RS face that interacts with the central pair. This may confer analogous electrostatic interaction with the central pair complex, implicated in beat control (Grossman-Haham et al., 2021). In contrast, TbRSP9L displays more differences in predicted structure to *C. reinhardtii* RSP9 and has a very short, albeit still acidic, loop (Fig. 1D).

**Fig. 2.**
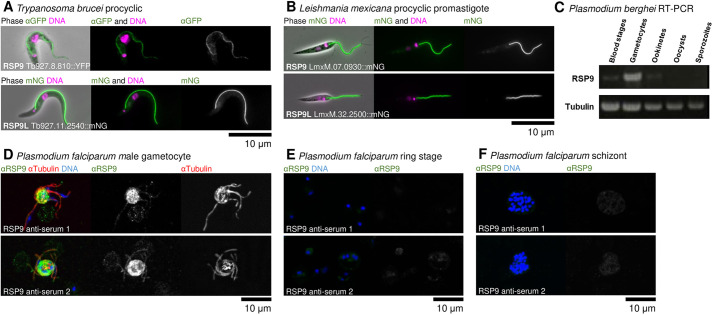
**RSP9 orthologues are flagellar proteins in *Trypanosoma*, *Leishmania* and *Plasmodium*.** (A) Representative immunofluorescence micrographs showing flagellar localisation of RSP9 (top) or RSP9L (bottom) by C-terminal tagging in procyclic *T. brucei*. Combined phase-contrast, DAPI or Hoechst 33342 (DNA stain) and YFP (an anti-GFP antibody) or mNG tagging, respectively. Representative of one experiments (additional replication is in Fig. S2). (B) Representative fluorescence micrographs showing flagellar localisation of RSP9 (top) and RSP9L (bottom) in *L. mexicana* promastigote. Combined phase-contrast, Hoechst 33342 (DNA stain) and tagged mNG RSP9 and RSP9L. (C) RT-PCR analysis of *P. berghei rsp9* mixed blood stages, gametocytes, ookinetes, oocysts and sporozoites. PCR using the constitutively expressed *tubulin* gene served as control for cDNA quality. (D) Representative immunofluorescence micrographs showing the flagellar localisation of RSP9 in *P. falciparum* male gametocytes using a specific anti-PfRSP9 antibody*.* Anti-RSP9 anti-serum 1 (top) or 2 (bottom) (anti-RSP9), anti-tubulin II and DAPI (DNA stain). (E,F) Representative immunofluorescence images show a weak RSP9 signal in ring stages (E) and schizonts (F), respectively. Combined RSP9 anti-serum 1 (top) or 2 (bottom) and DAPI (DNA stain).

First, we sought to determine whether *T. brucei* and *L. mexicana* RSP9 family proteins and *P. berghei* RSP9 are, indeed, flagellar proteins. In *T. brucei*, the genome-wide localisation project TrypTag showed that both RSP9 and RSP9L are axonemal proteins by N- and C-terminal tagging, respectively (Dean et al., 2017), which we replicated with YFP tagging in *T. brucei* (Fig. 2A) and mNeonGreen (mNG) tagging in *L. mexicana* (Fig. 2B). Tagged RSP9 and RSP9L signal was present near the basal body and continued to the end of the flagellum, indicating that the protein is located in the axoneme and not the para-axonemal structure called the paraflagellar rod (Halliday et al., 2019; Kohl et al., 1999).

In *P. berghei*, RT-PCR analysis showed that *rsp9* mRNA is present in mixed blood stages containing mainly asexual parasites, but also in activated gametocytes, and to a very low extent in ookinetes, but not in oocysts or sporozoites (Fig. 2C). GFP-tagging of PbRSP9 was unsuccessful after several attempts; however, anti-PfRSP9 antisera were available. We thus determined RSP9 localisation in the human parasite *P. falciparum* using two specific already available anti-PfRSP9 antisera. In exflagellating gametocytes, the anti-PfRSP9 antibody signal colocalised with the anti-α-tubulin II marker on the axoneme (Fig. 2D). No signal was seen in the blood stages (Fig. 2E,F). However, as the transcript was detected by RT-PCR in low amounts in mixed blood stages, we hypothesise that male gametocytes, like female gametocytes, in the blood may be stockpiling repressed transcripts (Mair et al., 2006) necessary for rapid gametogenesis.

### RSP9 and RSP9-like are both required for motility in *Trypanosoma* and *Leishmania*

To test RSP9 function in *T. brucei*, we generated tetracycline-inducible *TbRSP9^RNAi^*, *TbRSP9L^RNAi^* and *TbRSP9/9L^RNAi^* cell lines (simultaneously targeting both paralogues) by RNA interference (RNAi). Quantitative RT-PCR was used to determine the efficiency of the RNAi knockdown. *mre11*, *ODA7* and *tert* were identified as the three most stable reference genes (RGs) to be used for this study, and expression levels of *TbRSP9* and *TbRSP9L* were then normalised using the geometric mean of their transcript levels. For each group, three biological replicates were analysed (Fig. S5A–C). *TbRSP9* mRNA was reduced by ∼40% in both the induced *TbRSP9^RNAi^* and the combined *TbRSP9/9L^RNAi^* cell lines, while *TbRSP9L* mRNA was reduced by ∼70% in both the induced *TbRSP9L^RNAi^* and the combined *TbRSP9/9L^RNAi^* cell lines. Although this represents only limited knockdown efficiency, the reduction in structural proteins such as RSP9 or RSP9L could still compromise axonemal stability. Analysis of these cell lines was done with this caveat in mind.

To test RSP9 function in *Leishmania*, we produced three clonal deletion cell lines deleting both alleles of *Lmrsp9* (ΔLmRSP9), both alleles of *Lmrsp9L* (ΔLmRSP9L), or both alleles of both *Lmrsp9* and *Lmrsp9L* (ΔLmRSP9/9L). Complete loss of the respective open reading frames (ORFs) was confirmed using diagnostic PCR from purified genomic DNA from each cell line (Fig. S5D–F), confirming successful deletion.

**Fig. 3.**
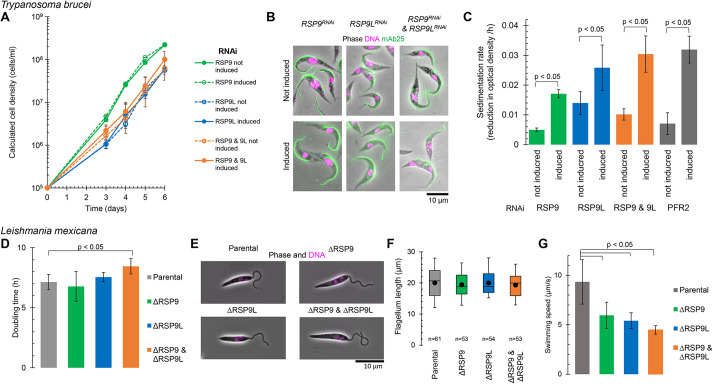
**Both RSP9 paralogues are necessary for motility in *T. brucei* and *L. mexicana.*** (A–C) Characterisation of RNA interference (RNAi) knockdown of RSP9 and RSP9L in *T. brucei*, using *TbRSP9^RNAi^*, *TbRSP9L^RNAi^* and *TbRSP9/9L^RNAi^* cell lines. (A) Cumulative growth curves under non-induced conditions (no tetracycline, solid lines) and induced conditions (+tetracycline, dashed lines). The data points show mean±s.d. (calculated from log-transformed cell density) for two, three or four clonal cell lines of *TbRSP9^RNAi^*, *TbRSP9L^RNAi^* and *TbRSP9/9L^RNAi^*, respectively. (B) Representative immunofluorescence fluorescence micrographs showing no morphological change in the induced RNAi cell lines. Combined phase-contrast, DAPI (DNA stain) and mAb25 (an anti-axoneme monoclonal antibody) images are shown. (C) Cell sedimentation rate, determined from decrease in culture optical density at 600 nm, relative to the positive control of PFR2^RNAi^ (Durand-Dubief et al., 2003). Mean±s.d. of *n*=4 different time intervals; all changes were statistically significant (unpaired two-tailed Student's *t*-test, *P*<0.05). Representative of three experiments. (D–G) Characterisation of RSP9 and RSP9L deletion in *L. mexicana*, using ΔLmxRSP9, ΔLmxRSP9L and ΔLmxRSP9/9L cell lines. (D) Doubling time in exponential culture in comparison to the parental (Cas9T7) cell line. Mean calculated from *n*=3, 24 h time intervals from one clonal cell line, error bars represent the s.d. Only ΔLmxRSP9/9L statistically significantly differed from the parental cell line (unpaired two-tailed Student's *t*-test, *P*<0.05). (E) Representative fluorescence micrographs showed no morphological change in the deletion mutant cell lines. Combined phase-contrast and Hoechst 33342 (DNA stain) images are shown. (F) Flagellum length in comparison to the parental cell line. *n* indicates the number of cells measured from one clonal cell line. The box represents the median and interquartile range, the whiskers represent the fifth and 95th percentile, and the point represents the mean. No changes were statistically significant (unpaired two-tailed Student's *t*-test, *P*>0.05). (G) Swimming speed in comparison to the parental cell line. Mean±s.d. of *n*=3 replicates. All changes were statistically significant (unpaired two-tailed Student's *t*-test, *P*<0.05).

We then wanted to know whether both RSP9 and RSP9L were required for motility and viability in trypanosomatids, and whether there was a functional redundancy between RSP9 and RSP9L. We therefore compared the phenotype of knockdown or deletion of RSP9 or RSP9L alone in comparison to the knockdown or deletion of both (Fig. 3). There was no significant reduction in cell growth in either species for any of these mutants (Fig. 3A,D). Morphology of both species, as seen by light microscopy, was also unchanged: the positions of nucleus and kinetoplast, as well as the overall cell shape, were unaffected. Importantly, there was no apparent flagellum assembly defect or notable change to the appearance of the flagellum in either species (Fig. 3B,E,F). RSP9 and RSP9L are therefore not vital *in vitro* for either species.

We studied the effect of absence of RSP9 proteins on motility. We carried out motility assays in *T. brucei* using a sedimentation approach and in *L. mexicana* by tracking cell movement by microscopy. *TbRSP9^RNAi^*, *TbRSP9L^RNAi^* and *TbRSP9/9L^RNAi^* all showed a decrease in motility as evidenced by increased sedimentation similar to the positive control, *TbPFR2^RNAi^* (Durand-Dubief et al., 2003) (Fig. 3C). The *L. mexicana* deletion cell lines also all showed decreased motility, as indicated by a decreased mean swimming speed, as previously described for immotile mutants (Beneke et al., 2019; Gorilak et al., 2021; Wheeler et al., 2015) (Fig. 3G). In both species, RSP9 and RSP9L are therefore independently necessary for normal motility, suggesting no functional redundancy.

### Absence of RSP9 causes defects in male gametogenesis in *Plasmodium* and impairs life cycle progression

To test RSP9 function in *P. berghei*, we generated two clonal knockout lines, *ΔPbrsp9* and *ΔPbrsp9-gfp*, in the constitutive *gfp*-expressing *P. berghei* (Janse et al., 2006b) (Fig. S5G). Gene deletion was confirmed by Southern blotting and diagnostic PCR (Fig. S5H–K).

**Fig. 4.**
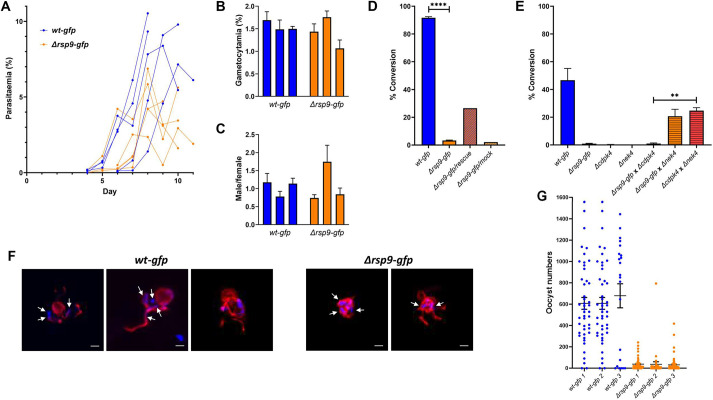
***P. berghei* RSP9 in asexual and sexual development.** (A) Asexual growth of *Δrsp9-gfp* and *wt-gfp* recorded from day 4–6 until day 11 post infection. (B,C) Gametocytaemia (B) and gametocyte sex ratio (C) were determined by infections of three mice each infected with *Δrsp9-gfp* or *wt-gfp*. Error bars represent s.e.m. (D) Ookinete conversion rate of *Δrsp9-gfp* and *wt-gfp* as well as *Δrsp9-gfp* transfected with rescue or mock plasmid. Error bars represent s.e.m. ****P<0.0001, determined by Welch's *t*-test. (E) *Δrsp9-gfp* and *wt-gfp* gametocytes crossed with either *Δcdpk4* (male deficient) or *Δnek4* (female deficient) strains. Error bars represent s.e.m. **P<0.01, determined by Welch's *t*-test. (F) Anti-α-tubulin staining microtubules of exflagellating *wt-gfp* and *Δrsp9-gfp* male gametocytes (red) with nuclei stained with DAPI (arrows). Scale bars: 2 µm. (G) Number of *Δrsp9-gfp* oocysts compared to *wt-gfp* oocysts in mosquito midguts. Bars depict mean with s.e.m.

To assess whether the gene deletion had an impact on asexual proliferation in the blood, mice were infected with a similar number of *Δrsp9-gfp* and *wt-gfp* parasites, and the parasitaemia was compared between the parental and mutant lines. During the first days after inoculation, no significant difference in parasite growth between control and *Δrsp9-gfp*-infected mice was observed (Fig. 4A). Intriguingly, in late stages of infection (day 9), when formation of gametocytes is more likely to occur, the parasitaemia of *Δrsp9-gfp* was significantly lower than that of *wt-gfp* (Fig. 4A). In contrast, gametocyte production (Fig. 4B) and sex ratio (Fig. 4C) were unaffected by the knockout of *Pbrsp9*. Therefore, RSP9 does not appear to be involved in gametocyte formation.

To assess the role of PbRSP9 in gamete formation and fertilisation, mixed blood stages from infected mice were allowed to undergo gametogenesis and fertilisation *in vitro.* Successful fertilisation and formation of ookinetes was quantified after immunostaining with anti-Pbs28 antibody as the ratio of mature ookinetes and retorts (immature ookinetes) to all Pbs28-positive cells (ookinetes and activated macrogametocytes). The lack of RSP9 almost completely abolished fertilisation and subsequent ookinete formation (Fig. 4D) but could be rescued by complementation with episomally expressed *rsp9* (Fig. S5J), showing that, indeed, the deletion of the *rsp9* gene resulted in a fertilisation phenotype (Fig. 4D). To investigate whether the loss of RSP9 affected male and not female gametocytes, genetic crossing using a male-defective line that produces normal female gametes was performed. The parasite line *Δcdpk4* (Billker et al., 2004) yielded similar numbers of ookinetes to *Δrsp9* alone, whereas fertilisation with the female-defective line *Δnek4* (Reininger et al., 2005) resulted in similar numbers of ookinetes to those obtained from a cross of *Δcdpk4* with *Δnek4* (Fig. 4E). This indicates that female *Δrsp9* gametocytes and gametes are fully functional, whereas male *Δrsp9* gametocytes, although formed, are severely impaired in their function.

To understand at which level male gametocytes or gametes were defective, we examined male gametogenesis in detail. The male gametes, *de facto* flagella each with an axoneme and associated nucleus, swim free from the gametocyte during a process called exflagellation. During this event, the male gametes beat vigorously to free themselves from the residual gametocyte body. The free beating flagella are clearly visible as wavy threads protruding from the round gametocyte body. Exflagellation events were never observed by light microscopy in *Δrsp9-gfp* parasites. We next investigated axoneme formation and DNA replication in activated male gametocytes by simultaneous staining with an anti-α-tubulin II antibody that specifically stains *Plasmodium* male gametocytes and gametes (Rawlings et al., 1992) as well as DAPI to visualise the eight newly replicated copies of the genome. At 15 min post activation, free WT microgametes were clearly stained, with both anti-α-tubulin II antibody and DAPI revealing a wavy structure typical of motile gametes. Nuclei are mostly associated with flagella, although, in some cases, nuclei can be observed residing or remaining in the main male gametocyte body, consistent with the prior observation that ∼50% of free gametes are anucleate in WT parasites (Sinden, 1975). Although *Δrsp9* show extensive staining with anti-α-tubulin II antibody and have clearly distinguishable nuclei, their flagella seem to protrude only partially. Most importantly, free male gametes were never observed to have a nucleus (Fig. 4F). Mitosis therefore appears to be completed normally, whereas subsequent axoneme motility appears to be abolished.

Despite the observed widespread impairment in male gamete formation, surprisingly, *Δrsp9* parasites are able to form a small number of ookinetes. To assess whether these ookinetes were capable of completing the life cycle, mosquitoes were fed on mice infected with *wt-gfp* or *Δrsp9-gfp* parasites, respectively, and oocyst numbers in the midguts were assessed (Fig. 4G; Table S5). The number of oocysts obtained in the *Δrsp9-gfp*-infected mice was significantly lower than that in the control line, correlating with the lower number of ookinetes produced. Mosquitoes infected with *wt-gfp* or *Δrsp9-gfp* parasites were also assessed for sporozoite loads, and these were shown to be very variable in both the WT and mutant line; additionally, infected mosquitoes could successfully transmit *Δrsp9-gfp* to naïve mice (Table S4).

Taken together, RSP9 was shown to be critical for male gamete release and thus fertilisation, but not for gametocyte activation. However, deletion of RSP9 did not have critical impact on any other aspect of the parasite’s life cycle.

### RSP9 is essential for RS head formation, which disrupts axoneme formation in *Plasmodium* but not *Trypanosoma* and *Leishmania*

**Fig. 5.**
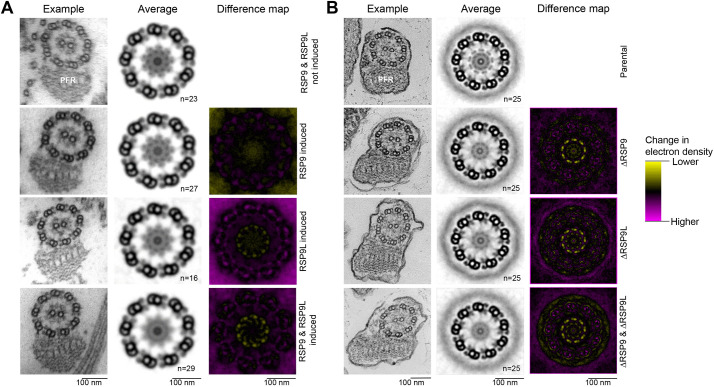
**Both paralogues of RSP9 are necessary for RS head, but not overall axoneme, assembly in *T. brucei* and *L. mexicana.*** (A) Ultrastructure change upon RSP9 and RSP9L RNAi in *T. brucei.* Transmission electron micrographs of transverse sections of detergent-extracted axonemes in induced *TbRSP9^RNAi^*, *TbRSP9L^RNAi^* and *TbRSP9/9L^RNAi^* cell lines in comparison to uninduced *TbRSP9/9L^RNAi^* cell lines. PFR, paraflagellar rod. (B) Ultrastructure change upon RSP9 and RSP9L deletion in *L. mexicana.* Electron micrographs of transverse sections of axonemes in ΔLmxRSP9, ΔLmxRSP9L and ΔLmxRSP9/9L in comparison to the parental cell line (Cas9T7). In A and B, the first column shows one representative axoneme cross-section. The second column shows an averaged axoneme structure, in which axoneme cross-sections have had perspective deviation from circularity corrected, followed by ninefold rotational averaging and averaging across multiple axoneme (*n* indicates the number of axonemes used). The third column shows an electron density difference map, resulting from subtraction of the induced RNAi or deletion mutant average axoneme image from the uninduced control or parental cell line. Yellow indicates more electron density in the uninduced control or parental cell line; magenta indicates more electron density in the induced RNAi or deletion mutant. There is a specific loss in electron density from the RS heads in the induced *TbRSP9L^RNAi^*, *TbRSP9/9L^RNAi^*, ΔLmxRSP9, ΔLmxRSP9L and ΔLmxRSP9/9L images.

To study the effect of gene knockdown or deletion on flagellar ultrastructure in *T. brucei*, *L. mexicana* and *P. berghei*, we analysed the control and mutant cell lines by electron microscopy. In *T. brucei* and *L. mexicana*, no large defects in the axoneme organisation could be observed (Fig. 5). The ninefold symmetry of the outer doublets and the central pair appeared unchanged in almost all cells, and some RSs could be seen. Given this lack of profound defect visible in single electron micrographs, we averaged the nine doublets in individual sections by ninefold rotation and subsequently used multiple axonemes to generate averaged views of the axoneme electron density (Fig. 5). The *TbRSP9^RNA^*^i^ cell line had no detectable loss of RS head electron density; both the *TbRSP9L^RNAi^* and *TbRSP9/9L^RNAi^* cell lines showed more loss of electron density at the RS heads than *TbRSP9^RNA^*^i^ (Fig. 5A). In contrast, all three *Leishmania* mutants (*Δ*LmxRSP9, *Δ*LmxRSP9L and *Δ*LmxRSP9/9L) had a clear loss of RS head electron density, with the *Δ*LmxRSP9/9L double-deletion mutant having comparable loss of electron density to the single-deletion *Δ*LmxRSP9 or *Δ*LmxRSP9L mutants (Fig. 5B). The RS stalk remained visible in all the mutants. Bearing in mind the limited RSP9 RNAi knockdown in *T. brucei*, we therefore conclude that RSP9 and RSP9L are both necessary for RS head assembly but not for overall axoneme organisation.

**Fig. 6.**
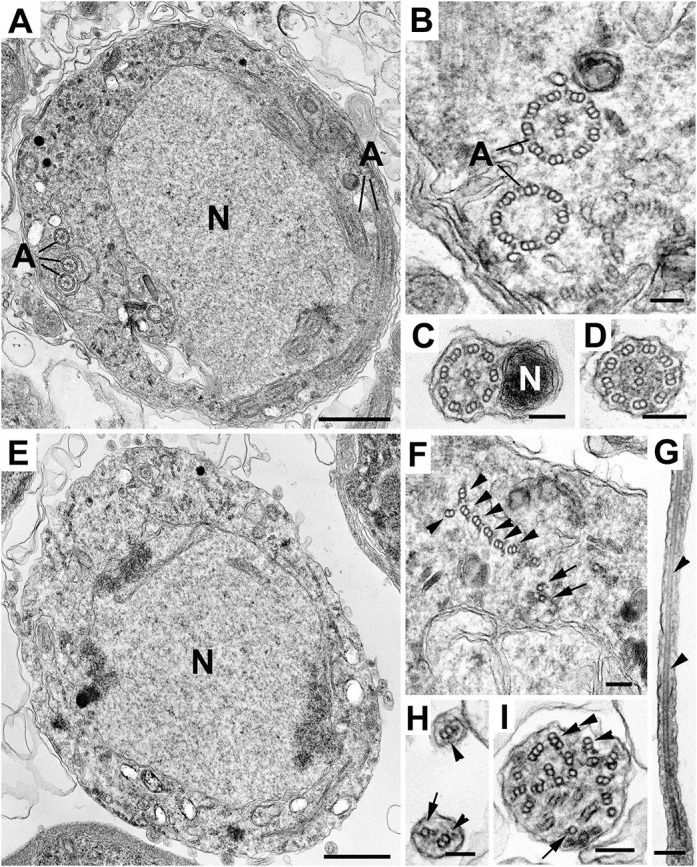
***Plasmodium* RSP9 is necessary for 9+2 axoneme formation and production of viable male gametes.** (A–I) Electron micrographs showing *wt-gfp* (A–D) and *Δrsp9-gfp* (E–I) male gametocytes and post-exflagellation microgametes. (A) Section through a mid-stage *wt-gfp* male gametocyte, illustrating the central nucleus (N) and the cytoplasm containing a number of cross-sectioned axonemes (A). (B) Detail of the cytoplasm of a stage similar to that in A, showing cross-sectioned axonemes. Note that one has the normal 9+2 axoneme whereas the other lacks the central pair (9+0). (C,D) Cross-sections through free microgametes, showing the classical 9+2 flagellar axoneme and the electron-dense nucleus (N). (E) Section of a late-stage *Δrsp9-gfp* male gametocyte, showing the central nucleus but the absence of axonemes within the cytoplasm. (F) Enlargement of a portion of cytoplasm similar to that in E, showing disorganised, but possibly connected, groups of doublet (arrowheads) and single (arrows) microtubules. (G–I) Longitudinal (G) and cross-sectioned (H,I) cytoplasmic process extending from microgametocytes, containing abnormal numbers and the disorganised arrangement of single (arrows), doublet (arrowheads) and even triplet (double arrowhead) microtubules. Note the absence of a nucleus. Scale bars: 1 µm (A,E) and 100 nm (B–D,F–I).

In *Plasmodium*, we analysed axonemal structures in WT and *Δrsp9* gametocytes. During microgametogenesis, multiple structural changes occur in the microgametocyte, such as threefold genome replication with an eightfold increase in the number of nuclear poles, chromosome condensation in the nucleus, and formation and growth of eight axonemes in the cytoplasm. Even in WT parasites, axoneme formation is frequently imperfect, with some axonemes lacking the central pair microtubules or possessing fewer outer doublets (Fig. 6A,B). In *Δrsp9* microgametocytes, the nucleus appeared similar to that in WT microgametocytes, but there were marked cytoplasmic differences. Axoneme assembly is profoundly defective: although numerous singlet and doublet microtubules were present, they appeared disorganised and randomly distributed throughout the cytoplasm (Fig. 6E,F). However, the electron-dense areas between the microtubule doublets may indicate that these doublets are still linked. Fully formed axonemes were very rare (observed only three times in 300 microgametocytes).

Male gametes in the process of being released can occasionally be observed by electron microscopy, as the axonemes and associated nuclei protrude then bud from the microgametocyte surface to form the free microgametes. In WT parasites, it was possible to identify free microgametes with a 9+2 flagellar structure and adjacent nucleus (Fig. 6C,D). In contrast, a thorough search of the RSP9 deletion mutant failed to identify any microgametes with a 9+2 structure. However, some structures protruding from the gametocyte surface contained microtubules, perhaps representing a partial exflagellation process as also seen by immunofluorescence (Fig. 4F, right panel). These structures contained variable numbers of singlet or doublet microtubules, with little evidence of the typical and regular axonemal organisation, and without an associated nucleus. Occasionally, triplet microtubules were observed, an arrangement that is normally not seen in any *Plasmodium* life cycle stages (Fig. 6G–I). These results, taken together with our observations by immunofluorescence (Fig. 4F), lead us to hypothesise that the disruption of the 9+2 axonemal structure or flagellar instability abolishes motility, impairing exflagellation and preventing recruitment of nuclei to a motile gamete, greatly reducing transmission efficiency.

## DISCUSSION

RSs are an essential part of the motile axonemal structure and are required for coordinated movement of the axoneme. The overall shape of RSs, as visualised by electron microscopy, is highly conserved across all ciliate organisms, and many *C. reinhardtii* RSP orthologues are present throughout the flagellate eukaryotes.

Fundamental understanding of RSPs is very largely derived from studies in *C. reinhardtii*, in which a large number of components were identified (Yang et al., 2006) and many mutants have been characterised: *pf1* (RSP4) and *pf14* (RSP3) (Luck et al., 1977; Piperno et al., 1977), *pf17* (RSP9), *pf24* (RSP2), *pf25* (RSP11) and *pf26* (RSP6) (Huang et al., 1981), as well as *fla14* (RSP22) (Pazour et al., 1999). These studies showed that RSPs are necessary for motility and that the canonical 9+2 axoneme arrangement is not dependent on RSPs; however, RS head assembly is dependent on stalk assembly.

The RSP1/10 and RSP4/6 families, along with RSP9 and RSP3, are the most widely conserved RSPs. Our comprehensive bioinformatic confirmed this, and showed that *Plasmodium* only has orthologues of RSP3, RSP4/6 and RSP9 showing conservation of only a few core RSPs. We note that, in the related organism *T. gondii*, orthologues of RSP16, RSP23 and RSP20 could additionally be detected, whereas *Eimeria* possesses only one additional RSP, namely RSP20. This is therefore likely to be an evolutionarily recent streamlining in *Plasmodium*, perhaps towards a minimal RS, contrasting the conservation of many RSPs in trypanosomatids. RSP9 orthologues, as expected for conserved RSPs, are required for flagellar motility in *Plasmodium* and trypanosomatid unicellular parasites. Unexpectedly, absence of RSP9 was demonstrated to cause a profound failure in axoneme assembly only in *Plasmodium.* It thus can be hypothesised that *Plasmodium* has evolved a minimal essential structure for the axoneme for which deletions can be lethal, whereas for most other organisms redundancy of axoneme components ensures stability even when mutations or deletions occur.

It is unknown whether axoneme assembly is similarly dependent on RSPs in other apicomplexans; to our knowledge, the molecular aspects of flagellar assembly and microgametogenesis have not been studied in other apicomplexans so far. The coccidian parasites *T. gondii* and *Eimeria tenella* have an extended repertoire of RPSs compared to *Plasmodium* (Fig. 1A) and also possess genes encoding IFT-related proteins (Van Dam et al., 2013). These parasites may have machinery or structures stabilising the axoneme, whereas *Plasmodium* is more prone to misassembly and disassembly (Depoix et al., 2020). One of the setbacks of rapid microgametogenesis is the limitation to three rounds of endomitosis and thus production of only eight microgametes per gametocyte. In contrast, coccidians can produce from a few tens to several hundreds over a period of hours to days (Ferguson et al., 1977, 1974, 1980). Although coccidian microgametes are produced within a protective parasitophorous vacuole inside an enterocyte, *Plasmodium* gametocytes, gametes, zygotes and eventually ookinetes must survive the intensely hostile environment of the mosquito midgut lumen. The processes from gametocyte activation to ookinete invasion of the mosquito gut wall must be rapid to ensure transmission. Therefore, extremely rapid but error-prone assembly of the simplest functional axoneme, and a low number of microgametes produced, may represent key adaptations of the parasite to its definitive host.

In trypanosomatids, previous analysis of RSP mutants showed similarities to *C. reinhardtii.* In *T. brucei*, RSP3 is required for normal motility and normal cytokinesis (to which motility contributes) (Ralston et al., 2006). Like the analogous *C. reinhardtii pf14* mutant (Diener et al., 1993), TbRSP3 RNAi knockdown caused total loss of the RS structure yet no disruption of the 9+2 organisation (Ralston et al., 2006). Furthermore, RSP4/6 deletion in *L. mexicana*, in a high-throughput screen, also resulted in defective swimming, but no large flagellum assembly defect by light microscopy (Beneke et al., 2019).

The first study of an RSP9 mutant was performed with the *C. reinhardtii pf17* mutant, which displayed reduced motility due to loss of the entire RS head (formed of RSP1, RSP4, RSP6, RSP9 and RSP10), but retained an axoneme structure with a central pair (Huang et al., 1981). Comparable phenotypes are seen in metazoan mutants: knockdown of the zebrafish orthologue of RSP9 (*rsph9*) gene disrupts ciliary motility, and some cases of primary ciliary dyskinesia in humans are linked with the *RSPH9* gene (Castleman et al., 2009; Frommer et al., 2015). Human *rsp9* mutations can prevent RS head assembly (Frommer et al., 2015), and homozygous knockout of the *rsp9* (also known as *Rsph9*) gene in mice leads to altered beating of the cilia bundles in the absence of a large impact in axonemal organisation (Zou et al., 2020). We analysed both *T. brucei* and *L.mexicana* RSP9 mutants. We demonstrate here that the two trypanosomatid RSP9 orthologues have similar functions, both of which are important for RS head assembly and motility, but not for overall structural stability of the 9+2 axoneme organisation. Owing to a limited knockdown of *T. brucei* RSP9 and RSP9L expression in the RNAi mutants, we base our conclusions mainly on the knockout data from *L. mexicana* and use the *T. brucei* results as confirmation of the phenotype. The presence of two RSP9 paralogues in *T. brucei* and *L. mexicana* reflects a wider pattern of gene duplications in RS evolution, likely linked with adaptation of the various symmetries in the RS head: in *C. reinhardtii*, there are two molecules of each of the RSP1/10 family, RSP4/6 family and RSP9 in each of the two RS head lobes. Our phylogenetic analysis indicates that *C. reinhardtii* RSP4 and RSP6 and trypanosomatid RSP9 and RSP9L are both likely to have resulted from lineage-specific gene duplication and divergence (Fig. S1). This suggests that trypanosomatid RSP9 and RSP9L may be acting as a heterodimer analogous to the *C. reinhardtii* RSP4–RSP6 heterodimer in each RS head lobe.

The *P. berghei* transcript for RSP9, a well-conserved RS head protein, was present not only as expected in activated gametocytes and as a weak signal in ookinetes, but also in mixed blood stages (Fig. 2C). Although in the ookinete preparation, a small amount of co-purification of male gametocytes that failed to exflagellate is probable, we could not determine whether the transcript in the mixed blood stages was derived from immature male gametocytes or asexual stages (stages that cannot be distinguished microscopically). A possible explanation is that the *rsp9* transcript is stockpiled for use in rapid exflagellation once reaching the mosquito midgut. Transcriptomic studies have shown that, indeed, the *rsp9* transcript is already present in male gametocytes (Lasonder et al., 2016; Yeoh et al., 2017). Alternatively, RSP9 could have a secondary function in asexual parasites. This is corroborated by our observation that deletion of *rsp9* resulted in reduced late-stage asexual proliferation in the intermediate host (Fig. 4A). Indeed, in *P. falciparum*, transcriptomics of synchronised ring stages revealed *Pbrsp9* mRNA associated with ring-stage polysomes indicative of the gene being actively translated (Bunnik et al., 2013), although contributions from gametocytes have not been assessed.

Although the lack of PbRSP9 results in impaired motility of axonemes during exflagellation and an aberrant microtubule structure, a few ookinetes are still formed that are capable of forming oocysts, which then produce infective sporozoites. This phenomenon has already been observed in *P. berghei* in the knockout of the basal body component SAS-6 (Marques et al., 2015; Depoix et al., 2020). If a few microgametes are released, random contact of male and female gametes may occur and lead to the formation of ookinetes.

Flagellar motility is critical for both the trypanosomatid and *Plasmodium* parasite life cycle. However, the details differ dramatically: although many trypanosomatid life cycle stages are flagellate and motile, with a flagellum that is retained over multiple multi-hour cell cycles, *Plasmodium* has a single, very short lived, but essential flagellar state typical for organisms that use flagella solely for sexual reproduction. Perhaps the *Plasmodium* axoneme benefits from a simpler RS architecture utilising a reduced, minimal, RSP repertoire for rapid assembly and tolerates a RS structure with less resilience because of the microgametes' short working life [30–60 min (Sinden and Croll, 1975)]. In the absence of redundancy, it is not surprising that any axonemal protein is critical. In contrast, it could also be hypothesised that *Plasmodium*, with a rather small, streamlined genome, dispenses wherever possible with complex structures and redundancies, which in the case of RSs result in a less stable, vulnerable to disruption, RS assembly as a trade-off. Alternatively, perhaps the vital role of RSP9 for *Plasmodium* axoneme assembly is linked with the mechanisms of the unusual rapid IFT-independent cytoplasmic assembly of this 14 µm structure in the ∼10 min between activation and exflagellation. In most organisms, RSs are partially assembled in the cytoplasm before IFT-dependent entry to the flagellum (Diener et al., 1993, 2011); the intracytoplasmic axoneme assembly in *Plasmodium* removes this constraint.

In this comparative study, we elucidate the localisation and roles of RSP9 proteins and orthologues in *T. brucei*, *L. mexicana* and *Plasmodium*. We find that, in trypanosomatids, in which all life cycle stages are flagellated, the RSP9 proteins are important for normal motility and RS head assembly. In contrast, *Plasmodium* RSP9 is an essential component of a minimal structure necessary for the mechanism of male gamete formation, in which gene loss reduced transmission significantly. Our results provide a foundation for better understanding of flagella in unicellular parasites and increased knowledge of RSs.

## MATERIALS AND METHODS

### Ethics statement

All experiments involving laboratory animals were performed in accordance with the European Union regulations ‘EU Directive 86/609/EEC’ and the regulations of the United Kingdom Animals (Scientific Procedures) Act 1986.

### Bioinformatic analysis

Predicted protein sequences for *Trypanosoma brucei brucei* TREU927 and *L. mexicana* MHOMGT2001U1103 were downloaded from TriTrypDB, and those for *P. falciparum* 3D7 and *P. berghei* ANKA were obtained via PlasmoDB, both projects within VEuPathDB (Amos et al., 2021). Predicted protein sequences for other eukaryotes were downloaded from UniProt.

Reciprocal best BLAST protein sequence searches were carried out using NCBI BLAST+ version 2.9.0+, and orthogroups were identified using OrthoFinder version 2.5.4 (using Diamond v2.0.5.143 and FastME 2.1.4) (Emms and Kelly, 2015, 2019). Protein structures were predicted using AlphaFold (Jumper et al., 2021; Mirdita et al., 2022; Wheeler, 2021), using the exact pipeline previously described (Wheeler, 2021). Phylogenetic trees were constructed using GenomeNet (https://www.genome.jp/), using MAFFT version 6.861 for sequence alignment and PhyML for maximum likelihood tree construction, reporting approximate likelihood ratio test χ^2^ parametric values for branch supports.

### Parasite cell lines, maintenance and culturing

*T. brucei* cell lines used in this study are derivatives of *T. brucei brucei* strain 427 and cultured at 27°C in SDM-79 medium (Brun and Schönenberger, 1979) (PAA Laboratories, ME090164P1) supplemented with 2.5 mg/ml hemin (Sigma-Aldrich) and 10% foetal calf serum (PAA Laboratories). All selection drugs for cell culture were purchased from Invivogen (France). Lack of contamination was confirmed by fluorescent microscopy with a DAPI DNA stain.

Cas9T7 *L. mexicana* derived from WHO strain MNYC/BZ/62/M379, expressing Cas9 and T7 RNA polymerase (Beneke et al., 2017), were grown in M199 (Life Technologies) supplemented with 2.2 g/l NaHCO_3_, 0.005% hemin, 40 mM HEPES·HCl (pH 7.4) and 10% FCS. *L. mexicana* cultures were grown at 28°C. Culture density was maintained between 1×10^5^ and 1×10^7^ cells/ml for continued exponential population growth. Culture density was measured using a haemocytometer. Identity was confirmed by recent mRNA and genomic sequencing, and lack of contamination was confirmed by fluorescent microscopy with a Hoechst 33342 DNA stain.

*P. berghei gfp*-expressing strain 507 cl1 (Janse et al., 2006b) was maintained as cryopreserved stabilates and by cyclic blood passage in 6- to 8-week-old female Tuck-Ordinary mice (Harlan Laboratories, UK). Hyper-reticulocytosis was induced by intraperitoneal treatment of mice with 200 µl 6 mg/ml phenylehydrazine (PH; BDH Chemicals Ltd, UK) 2–3 days prior to parasite inoculation. Schizonts and ookinetes were cultured as previously described (Ramakrishnan et al., 2013). Lack of contamination was confirmed by fluorescent microscopy with a DAPI DNA stain.

### Genetic modifications and validation of the mutants

In *T. brucei*, a fragment of each gene was chemically synthesised by GeneCust (Luxembourg), allowing the knockdown of the expression of TbRSP9 (Tb927.8.810), TbRSP9L (Tb927.11.2540) and the combined RSP9/9L. For RSP9 knockdown, the fragment was of 411 bp length (position 515–927 bp); for RSP9L knockdown, the fragment was of 338 bp (position 393–730 bp). For the combined knockdown of RSP9/RSP9L, a hybrid fragment was synthesised composed of 203 bp RSP9L fused with 216 bp of RSP9, position 60–262 bp of the *rsp9-like* gene sequence and 311–526 bp of the *rsp9* gene sequence. All products were sub-cloned in the pZJM vector (Morris et al., 2001; Wang et al., 2000), and the constructs were transfected in 13–29 cells that express the T7RNA polymerase and the tetracycline (TET) repressor (Wirtz et al., 1999) using the Amaxa nucleofector (Lonza). Cells were grown in the presence of 25 µg/ml hygromycin, 15 µg/ml neomycin and 2.5 µg/ml phleomycin. Double-stranded RNA production was induced by addition of 1 µg/ml tetracycline, and fresh tetracycline was added at each dilution.

For localisation, in *T. brucei*, the first 499 bp of the *rsp9* gene were cloned in frame behind the eYFP gene in the p2675 vector (Kelly et al., 2007). After linearisation within the *rsp9* gene sequence by BbsI (NEB), the constructs were transfected into WT cells. Integration into the endogenous *rsp9* locus resulted in a reconstructed complete YFP::RSP9 fusion. Resistant clones were selected by addition of 1 µg/ml puromycin (Invitrogen).

The mutants were validated by quantitative RT-PCR. Total RNA extraction was carried out using a Nucleospin RNA II kit combined with the Nucleospin RNA/DNA Buffer Set (Macherey Nagel). An additional step of gDNA digestion was performed using rDNAse Set (Macherey Nagel), followed by a purification step using clean-up protocol of the kit Nucleospin RNA XS (Macherey Nagel), according to the manufacturer's instructions. Purified RNA was quantified using a NanoDrop 2000 Spectrophotometer (Thermo Fisher Scientific); the A260/A280 ratio of the RNA samples was 2.146±0.026 (mean±s.d.), indicating the absence of protein, and its integrity was checked on 1% agarose gel (data not shown).

First-strand cDNA was synthesised from 0.5 µg total RNA using RevertAid H Minus Reverse Transcriptase (Thermo Fisher Scientific) with 0.2 µg of random hexamers in a 20 µl reaction mixture, according to the manufacturer's instructions. For each sample, a no-reverse transcription control was done in parallel for further assessment of gDNA contamination. Real-time PCR was performed on a LightCycler 480 (Roche Diagnostics France). Each reaction was performed in duplicate and contained 5 µl of SensiFAST™ SYBR^®^ No-ROX Kit Master Mix (Meridian Bioscience), 500 nM of each primer and 4 µl of 1:15 diluted cDNA sample, in a final volume of 10 µl. We included a non-template control (NTC) for each gene. The cycling conditions were as follows: one cycle at 95°C for 2 min, followed by 45 cycles at 95°C for 5 s, 60°C for 10 s, and 72°C for 12 s. The critical cycle (C_q_) was automatically calculated using the ‘second derivative maximum method’. At the end of the amplification, the melting temperature of the product was also determined using a melting curve program. For each pair of primers, the reaction efficiency was determined using a threefold serial dilution of cDNA as template performed in triplicate, covering a range of magnitude of 3 log_10_ and including the interval for the target being quantified. The standard curve was obtained by plotting C_q_ values against logarithm of dilutions. The efficiency of the reaction (*E*) was calculated from the slope value of the standard curve, as follows:
(1)

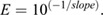


To identify suitable RGs for normalisation, five candidates [*Actin*, *Aldolase* (*aldo*), *mre11*, *oda7* and *tert*] were selected among genes for which expression stability had previously been evaluated in *Trypanosoma* (Brenndörfer and Boshart, 2010; Rotureau et al., 2014a). Primer sequences were designed with Primer-BLAST software (http://www.ncbi.nlm.nih.gov/tools/primer-blast/), except for genes *mre11* and *tert*, for which primer sequences were obtained from Brenndörfer and Boshart (2010). Specificities were checked for all primer pairs using Primer-BLAST and controlled using melting curves and migration of the amplicons in 3.5% high-resolution agarose electrophoresis. Genomic DNA contamination was assessed on minus reverse transcriptase controls with the Actin primer pair. No significant amount was found as C_q_ values were higher than their positive counterpart by at least 15 cycles.

For the five candidate RGs, C_q_ values obtained from the samples were analysed using GeNorm algorithms to analyse the expression stability, and to determine the optimum number of RGs required for an accurate normalisation. Expression levels (*Q*) of TbRSP9 and TbRSP9L were first expressed as fold changes of each sample relative to one day (*D*) 0 sample, using the formula
(2)

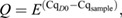
where *E* is the efficiency of the PCR reaction, and then normalised using the geometric mean of the expression levels of the validated RGs (Vandesompele et al., 2002). MRE11, ODA7 and Tert were identified as the three most stable candidate RGs to be used for this study and were therefore used as RGs. Gene IDs and primer sequences are presented in Table S2. Amplicon length, quantitative PCR efficiencies and *R*^2^ of the calibration curve are presented in Table S3.

For studies in *L. mexicana*, constructs and sgRNA templates for endogenous mNG-tagging templates were generated by PCR, as previously described (Beneke et al., 2017), and were transfected as previously described (Dean et al., 2015). The pLrPOT series of vectors was used as PCR templates for generating tagging constructs, specifically pLrPOT mNG Neo. Constructs and sgRNA templates for ORF deletion were generated by PCR and transfected as previously described, using pT Blast, pT Puro and pT Neo as templates (Beneke et al., 2017). Primers were designed using LeishGEdit (www.leishgedit.net/) (Beneke et al., 2017). Transfectants were selected with the necessary combination of 20 μg/ml puromycin dihydrochloride, 5 μg/ml blasticidin S hydrochloride and 40 μg/ml G-418 disulfate.

To verify loss of the target ORF in drug-resistant transfectants, a diagnostic PCR was performed by amplifying a short PCR product (100–300 bp) within the ORF of the target gene (primers are unique to the target gene). In the same diagnostic PCR, we use a positive control with a genomic DNA from the parental cell line to detect the target ORF. A further technical control PCR is required to demonstrate presence of DNA from the genomic DNA of the putative knockout cell line [e.g. amplify a short fragment from the paralysed flagella (PF16: LmxM.20.1400)].

To generate a loss-of-function mutant in *P. berghei*, the 5′ and 3′ untranslated region (UTR) of *rsp9* were cloned into pOB90 (courtesy of Oliver Billker, Umeå University, Umeå, Sweden), each gene fragment flanking the pyrimethamine resistance marker *tgdhfr-ts*. The 5′ UTR was amplified using 1-5UTRF and 2-5UTRR, and the 3′ UTR was amplified using 3-3UTRF and 4-3UTRR, amplifying 955 or 985 bp from genomic DNA, respectively. The PCR products of the 5′ UTR and 3′ UTR were digested with KpnI and ApaI or BamHI and XbaI and cloned into KpnI and XbaI-digested pOB90.

To obtain deletion mutants, plasmid PbRsp9ko was digested with KpnI and XbaI, and the drug selection cassette with the gene-specific flanking regions was extracted from an agarose gel. The DNA was precipitated and 2–3 µg was transfected into *P. berghei* schizonts cultivated and enriched as previously described (Ramakrishnan et al., 2013). The DNA was electroporated into schizonts using programme U33 on the Nucleofector electroporator (Amaxa GmbH) exploiting established protocols (Janse et al., 2006a). Electroporated parasites were allowed to re-invade blood from mice with induced hyper-reticulocytosis 2–5 days prior to transfection. The parasites and blood mixture was then injected intraperitoneally into naïve mice. One day post infection, mice received drinking water containing 70 µg/ml pyrimethamine to select transgenic parasites. Parasites were passaged into a naïve mouse a few days after the release of drug pressure and underwent a second round of drug selection. Clonal transgenic lines were obtained by limiting serial dilution. Correct integration was confirmed first by Southern blotting. A probe was amplified from WT gDNA using primers SouprobeF and SouprobeR (Table S2). The gDNA of *Δrsp9* and background lines were subjected to restriction digest by with NdeI. Genomic DNA was extracted from mixed, purified *Plasmodium* blood stages using the Wizard Genomic DNA Purification Kit (Promega). For Southern blotting, the DNA was digested with BstBI and separated on 0.8% agarose gels. The DNA was then transferred to Hybond-N+ (GE Healthcare, UK) nylon membrane and hybridised to the α^32^P-dATP-labelled probe using a High Prime DNA Labelling Kit (Roche) at 68°C using standard procedures (Sambrook et al., 1989).

Diagnostic PCRs were performed with gDNA using primers g and h to detect an intact WT locus, c and d to identify the *tgdhfr/ts* cassette, and a/b and e/f to probe for the integrated resistance cassette at the 5′ and 3′ end, respectively (Fig. 4G).

For the generation of a *Δrsp9* complementation construct, a 2913 bp fragment encompassing the 5′ UTR to the 3′ UTR was amplified using primers Rsp9koRes-F and Rsp9koRes-R from *P. berghei* ANKA 234 genomic DNA. Using ligation-independent In-Fusion cloning (TaKaRa), the PCR product was cloned into KpnI and EcoRI digested pOB277 (kindly provided by O. Billker) to yield plasmid pRsp9koRes. To generate a complemented parasite strain, *Δrsp9-gfp* schizonts were cultured as described (Ramakrishnan et al., 2013) and transfected with 5 µg of pRsp9koRes or a mock plasmid [Pbkin8B-gfp (Depoix et al., 2020)] as described above. The day after, parasites were subjected to drug selection for 7 days: mice were injected intraperitoneally with 2 mg/ml WR99210 (Jacobus Pharmaceuticals, USA) in 50% DMSO/50% PBS. Parasites were genotyped by diagnostic PCR using primers i and j (with primers for Pbs25 as control) and subsequently re-selected with WR99210 3 days post infection once.

Total RNA was extracted from various *P. berghei* life cycle stages using Trizol (Invitrogen, UK) and chloroform and treated with TURBO DNase from the TURBO DNA-*free*™ Kit (Invitrogen, UK). First-strand synthesis was performed using oligo(dT)_12-18_ (Invitrogen, UK) and the Moloney murine leukaemia virus reverse transcriptase (Invitrogen, UK). PCRs were performed using 54°C annealing temperature to amplify the *rsp9* transcript using RTrsp9F and RTrsp9R (Table S2). As a control, α-tubulin was amplified with RTαtubF and RTαtubR (Table S2).

### Assessment of asexual blood stage proliferation and gametocyte production and sex ratio

Naïve Tuck-Ordinary mice were infected intraperitoneally with 1000 *Δrsp9-gfp* or *wt-gfp* parasites for assessment of asexual growth. Three mice per strain and per experiment were used. Giemsa-stained blood smears were analysed from the onset of parasitaemia using three counts of 1000 red blood cells per mouse. Data were compared using Welch's *t*-test.

### *P. berghei* ookinete culture and crossing experiments

Tuck-Ordinary mice were treated with PH as above 3 days prior to infection by serial blood passage. For ookinete culturing, mixed blood stages were harvested by cardiac puncture of the mice and transferred to ookinete culture medium as previously described (Ramakrishnan et al., 2013). To obtain crosses of two different strains, mixed blood stages were mixed 1:1. To assess conversion of gametocytes to ookinetes, 24 h post inoculation, parasites were incubated with anti-Pbs28 antibody [monoclonal antibody 13.1 (Winger et al., 1988)] conjugated to Cy3 (Tewari et al., 2005). The proportion of mature ookinetes and retorts to all 13.1-positive cells (unfertilised macrogametocytes, ookinetes and retorts) was assessed by microscopy. All experiments were performed in triplicate.

### Assessment of exflagellation capacity and α-tubulin staining of male gametocytes

Before setting up ookinete cultures or mosquito infections, exflagellation was assessed as previously described (Ramakrishnan et al., 2013). To stain flagella with anti-α-tubulin, samples were fixed 15 min after inducing exflagellation in fresh 4% paraformaldehyde (PFA). The suspension was allowed to adhere onto poly-L-lysine-coated slides overnight at 4°C. The slides were washed once with PBS, and parasites were stained using the mouse monoclonal anti-α-tubulin II antibody (Sigma-Aldrich, T9026, 1:250). Secondary antibody Alexa Fluor 568-conjugated anti-mouse IgG for fluorescence detection was used at 1:1000 (Molecular Probes, Thermo Fisher Scientific, A-11008). The slides were mounted in Vectashield with DAPI (Vector Laboratories). Parasites were visualised on a Leica SP5 confocal microscope and acquired and analysed with the LAS AF Lite software (Leica).

### Assessment of oocyst loads and sporozoite loads in *Anopheles stephensi*

*A. stephensi* mosquitoes were reared as previously described. Mice treated with PH 3 days prior to infection were inoculated with either 507 cl1 or *Δrsp9-gfp*. To infect mosquitoes, 3- to 8-day-old female adult *A. stephensi* were starved overnight and subsequently allowed to feed on anaesthetised infected mice for 20 min at day 3 post infection. The day after feeding, mosquitoes that did not feed were removed. Mosquitoes were dissected for the quantification of oocyst loads in midguts 12 days post infection. Fluorescent microscopy was performed with the GFP channel and counted using Icy software version 1.5.1.0 (https://icy.bioimageanalysis.org/); statistical analysis was done using GraphPad Prism (Mann–Whitney *U*-test). Experiments were performed in triplicate. To assess infectious sporozoites, salivary glands from infected *A. stephensi* were removed 21 days post infection. Salivary glands from ten mosquitoes were pooled and homogenised in PBS, and sporozoites were estimated using a Neubauer haemocytometer.

### Mouse infections by mosquito biting (bite backs)

Tuck-Ordinary mice were treated with PH as above and infected by blood passage with *P. berghei*. *A. stephensi* mosquitoes were then fed 3 days post inoculum directly on infected mice after overnight starvation. These mosquitoes were maintained by sugar feeding and, at 21 days post infection, allowed to blood feed on C57Bl/6 mice. Blood-fed mosquitoes were counted and isolated. A subset of mosquitoes (fed or unfed) was selected for determination of salivary gland sporozoite load. To this end, salivary glands were removed, homogenised in PBS and counted using a Neubauer chamber. Parasitaemia of infected mice was assessed at day 6 post infection. Experiments were performed in triplicate.

### Microscopy

Immunofluorescence analysis of procyclic cells’ *T. brucei* was realised as previously described (Absalon et al., 2007; Demonchy et al., 2009). Briefly, cells were centrifuged, rinsed once and resuspended in 1× PBS, then spread out on glass slides and air dried. They were then fixed in methanol for 5 min at −20°C. After rehydration, cells were incubated for 60 min with the anti-GFP rabbit antiserum (Invitrogen, A-11122, 1:400 in PBS–1% bovine serum albumin) and then the anti-axoneme antibody mAb25 (1:20; Pradel et al., 2006). After three wash steps in PBS, the secondary antibodies were added (for YFP stain: goat anti-rabbit Alexa Fluor 488, Thermo Fisher Scientific, France, A-11008, 1:200; for mAb25: goat anti-mouse Alexa Fluor 488, Thermo Fisher Scientific, France, A-11001, 1:200). After 45 min of incubation, slides were washed three times and DNA was stained using DAPI (5 µg/ml). Cells were washed once with PBS. Slides were mounted in ProLong Gold Antifade Mountant (Thermo Fisher Scientific) and observed on a DMR Leica microscope using a 100× PL Fluotar objective. Images were captured with a Cool Snap HQ camera (Roper Scientific). Images were analysed using ImageJ (National Institutes of Health) and prepared using Adobe Photoshop (CS6).

*L. mexicana* expressing fluorescent fusion proteins were imaged live. Cells were washed three times by centrifugation at 800 ***g*** followed by resuspension in PBS. DNA was stained by including 10 μg/ml Hoechst 33342 in the second washing. Washed cells were settled on glass slides and were observed immediately. Widefield epifluorescence and phase-contrast images were captured using a Zeiss Axioimager.Z2 microscope with a 63×/1.40 numerical aperture (NA) oil immersion objective and a Hamamatsu ORCA-Flash4.0 camera. Cell morphology measurements were made in ImageJ (Collins, 2007).

For the localisation study of RSP9 in *Plasmodium*, anti-RSP9 antibodies were generated against *P. falciparum* RSP9, and one rabbit each was immunised with either peptide 1 (CRYPKNLTYDKIKNYN) or peptide 2 (EIQSDSYNRKGKK) (Eurogentec, UK). Antibodies from polyclonal serum were affinity purified using the corresponding peptides coupled to cyanogen bromide-activated sepharose (Sigma-Aldrich, UK). To validate the antibodies, erythrocytes from a sample of activated *P. falciparum* gametocytes were lysed with 0.05% saponin for 10 min and then washed with PBS. Parasites were then lysed in Laemmli buffer without SDS or reducing agent and separated with a polyacrylamide gel without SDS for native PAGE. Proteins were transferred onto nitrocellulose by semi-dry blotting (Trans-Blot TurboTransfer System, Bio-Rad) followed by a wash in TBS–Tween 20 and 1 h incubation in blocking buffer (5% bovine serum albumin in TBS–Tween 20). The blots were incubated overnight at 4°C in blocking buffer containing either serum 1 or 2 at 1:1000 dilution. After three washes with TBS–Tween 20, the blots were incubated with an 1:10,000 anti-mouse antibody conjugated to horseradish peroxidase. Following another three washing steps with TBS–Tween 20, detection was performed using Immobilon Western reagents (Millipore) and imaged with the ChemiDoc MP imager from Bio-Rad (Fig. S6). Following validation, *P. falciparum* gametocytes 15 min post activation, ring stages or schizonts were fixed in 4% PFA/PBS and permeabilised with 0.2% Triton X-100/PBS. Unspecific epitopes were blocked with 10% goat serum/3% bovine serum albumin/PBS. Parasites were stained using purified antibodies from sera 1:100 together with anti-α-tubulin antibody (Sigma-Aldrich, T9026, 1:250) and labelled with 1:1000 secondary anti-rabbit Alexa Fluor 488 (Molecular Probes, Thermo Fisher Scientific, A-11008) and anti-mouse Alexa Fluor 568 (Molecular Probes, Thermo Fisher Scientific, A-11004). Samples were mounted in Vectashield containing 4′,6-diamidino-2-phenylindole (DAPI; Vector Labs). Parasites were imaged using the Leica SP5 confocal microscope and analysed with LAS AF Lite Software (Leica).

### Motility assays

For motility analysis in *T. brucei*, a sedimentation assay was conducted as previously described by Ralston et al. (2006). 5×10^6^ uninduced and 6 days induced trypanosomes were incubated at 27°C in 1 ml medium in spectrophotometric cuvettes, and optical density was measured every 2 h compared to that in controls in which cells were resuspended. In *L. mexicana*, swimming behaviours are analysed for cells in the exponential growth phase in normal culture medium essentially as previously described (Wheeler, 2017). For cell swimming analysis, a 25.6 s video at five frames/s under darkfield illumination was captured from 5 μl of cell culture in a 250 μm deep chamber using a Zeiss Axioimager.Z2 microscope with a 10×/0.3 NA objective and a Hamamatsu ORCA-Flash4.0 camera. Particle tracks were traced automatically, and mean cell speed, mean cell velocity and cell directionality (the ratio of velocity to speed) were calculated as previously described (Beneke et al., 2019).

### Transmission electron microscopy

Uninduced and 6 days induced *T. brucei* (whole cells and detergent-extracted cytoskeletons) were prepared as previously described (Demonchy et al., 2009). 60 nm-thick sections were cut using an Ultramictome RMC Powertome XL and stained with 2% uranyl acetate. Grids were analysed on a Hitachi HT-7700 transmission electron microscope with a Hamamatsu digital camera.

For transmission electron microscopy, *L. mexicana* were fixed directly in medium for 10 min at room temperature in 2.5% glutaraldehyde (glutaraldehyde 25% stock solution, EM grade, Electron Microscopy Sciences). Centrifugation was carried out at room temperature for 5 min at 16,000 ***g***. The supernatant was discarded, and the pellet was fixed in 2.5% glutaraldehyde and 4% PFA (16% stock solution, EM grade, Electron Microscopy Sciences) in 0.1 M cacodylate buffer (pH 7.2) and contrasted with OsO_4_ (1%) (osmium tetroxide 4% aqueous solution, Taab Laboratories Equipment). After serial dehydration with ethanol solutions, samples were embedded in low-viscosity resin Agar 100 (Agar Scientific, UK) and left to polymerise at 60°C for 24 h. Ultrathin sections (70–80 nm thick) were collected on nickel grids using a Leica EM UC7 ultra microtome and stained with uranyl acetate (1%, w/v) (uranyl acetate dihydrate, Electron Microscopy Sciences) and lead citrate (80 mM, buffer made in-house). Observations were made on a Thermo Fisher Scientific Tecnai12 transmission electron microscope with a Gatan OneView camera.

Mixed blood stages of *P. berghei* were grown in mice after inducing hyper-reticulocytosis before infection as described above. Gametocytes were purified by re-suspension of infected whole blood in coelenterazine loading buffer (CLB; 20 mM HEPES, 20 mM glucose, 4 mM sodium bicarbonate, 1 mM EDTA, 0.1% bovine serum albumin in PBS, pH 7.25). The suspension was centrifuged at 600 ***g*** for 5 min, and all but some supernatant was removed in which the pellet was re-suspended. The parasite suspension was layered on top of a 48% Nycodenz cushion, which was spun for 10 min at 1000 ***g*** with acceleration and brake set to 0. The interphase was collected and washed in CLB and subsequently fixed in 4% glutaraldehyde in 0.1 M phosphate buffer and processed for routine electron microscopy as described previously (Ferguson et al., 2005). In summary, samples were post fixed in osmium tetroxide, treated en bloc with uranyl acetate, dehydrated and embedded in Spurr's epoxy resin. Thin sections were stained with uranyl acetate and lead citrate prior to examination in a JEOL12EX electron microscope.

### Ninefold rotational averaging of *T. brucei* and *L. mexicana* axonemes

Ninefold rotational averages of the axoneme structure (Markham rotations) were generated following perspective correction to ensure a circular axoneme cross-section, as previously described (Gadelha et al., 2013). Axoneme cross-sections were pooled from negative controls and then were stacked and averaged in ImageJ (National Institutes of Health) (Collins, 2007) to generate average proximal and distal axoneme electron density.

## Supplementary Material

10.1242/joces.260655_sup1 Supplementary information
