# Supplementary material for: Radial spoke protein 9 is necessary for axoneme assembly in Plasmodium but not in trypanosomatid parasites
Source: J Cell Sci. 2023 Jun 8;136(11):jcs260655. doi: 10.1242/jcs.260655 (PMC10309588; doi:10.1242/jcs.260655)
Supplement: Supplementary information [file joces-136-260655-s1.pdf]

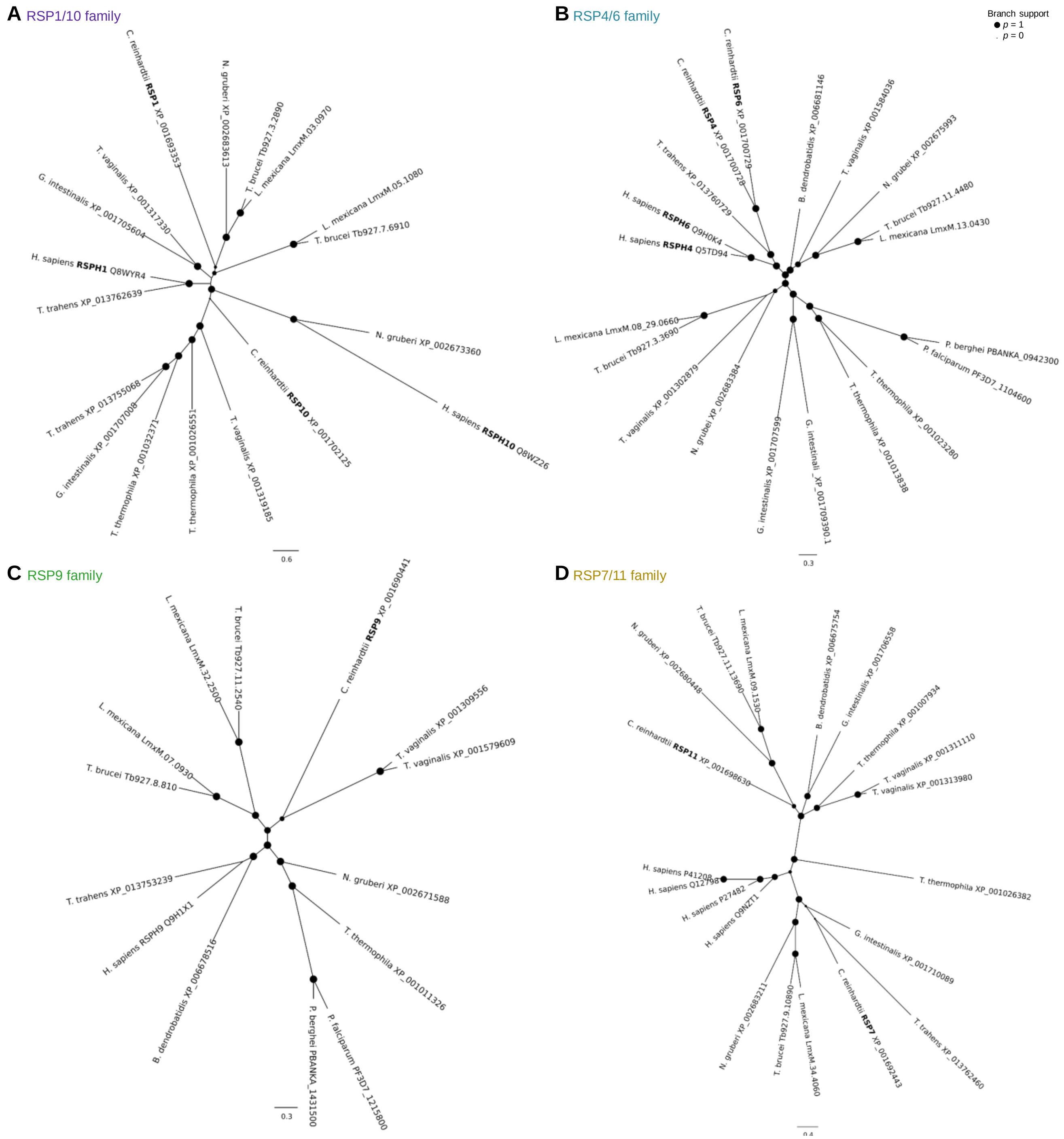

Fig. S1. Phylogenetic trees of the RSP1/10, RSP4/6, RSP9 and RSP7/11 families

Maximum likelihood phylogenetic trees constructed for *Batrachochytrium dendrobatidis, Chlamydomonas reinhardtii, Giardia intestinalis, Homo sapiens, Leishmania mexicana, Naegleria gruberi, Plasmodium berghei, Plasmodium falciparum, Tetrahymena thermophila, Thecamonas trahens, Trichomonas vaginalis* and *Trypanosoma brucei* orthologs of **A.** RSP1/10, **B**. RSP4/6, **C**. RSP9 and **D.** RSP7/11 families of RSPs. Leaf labels show species, accession number and, for *C. reinhardtii* and *H. sapiens*, gene names. Circles at the branch nodes indicate χ^2 approximate likelihood ratio test p values.

Journal of Cell Science • Supplementary information

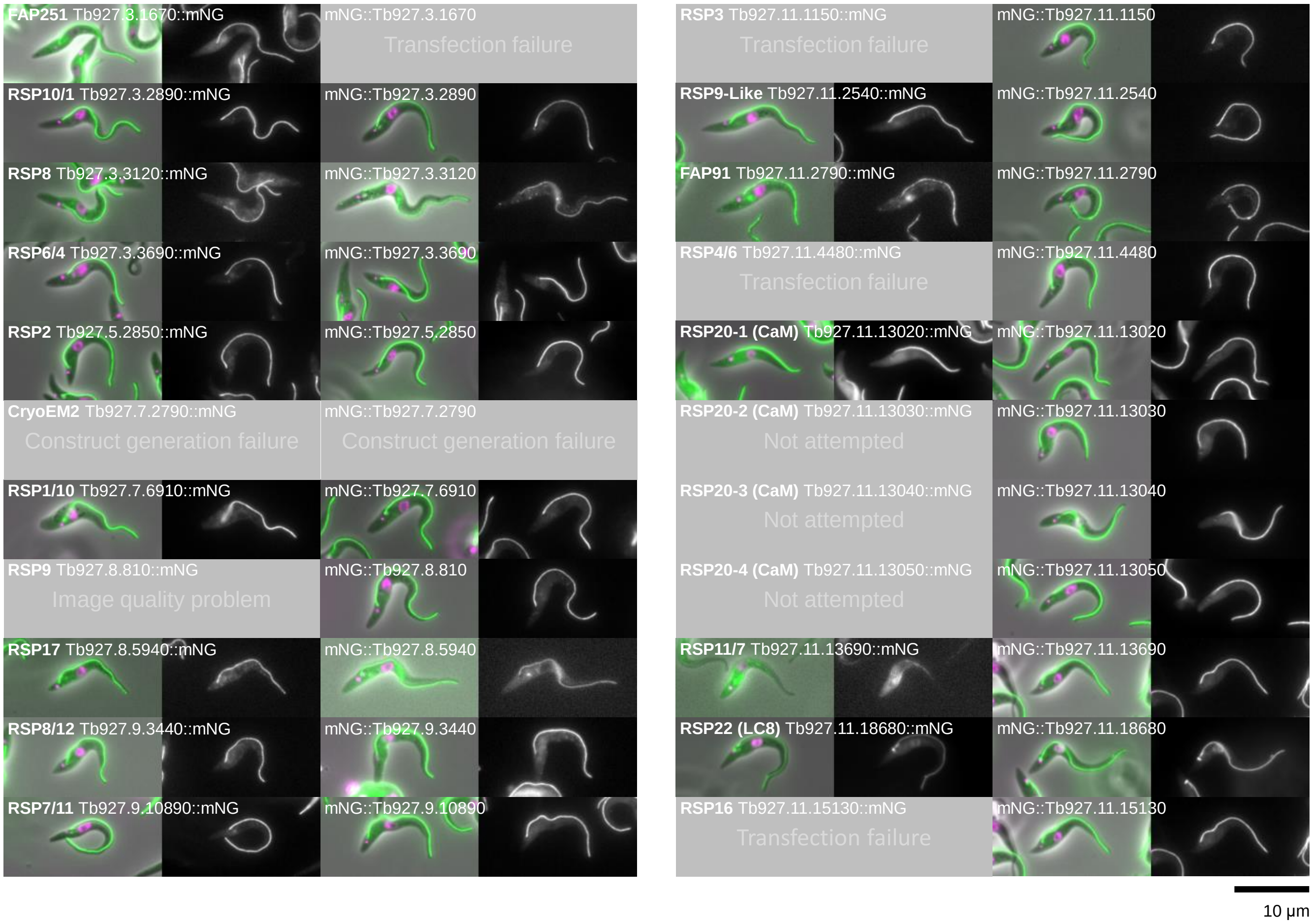

Fig. S2. Localisation of RSP candidates in *T. brucei* from a genome-wide protein localisation project Fluorescence micrographs of the sub-cellular localisation of RSP candidates as determined by N (left column) or C terminal tagging (right column) by the genome wide protein localisation project TrypTag (Dean *et al.*, 2017). Combined phase contrast (grey), Hoechst 33342 (DNA stain, magenta) and mNG tagging (green) respectively are shown.

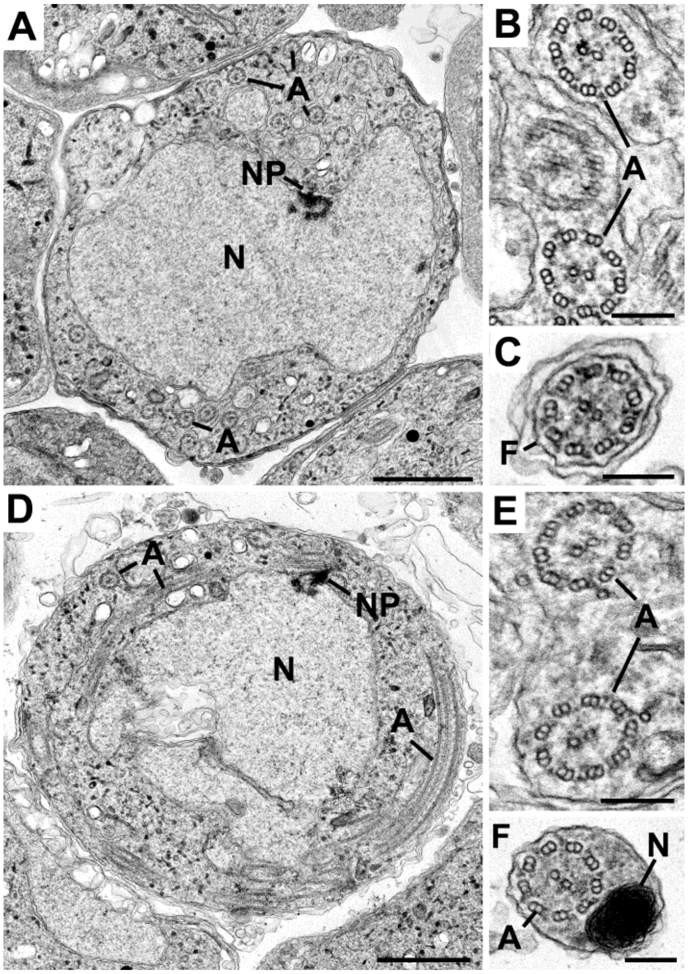

Fig. S3. Electron micrographs of sections illustrating microgametocytes of wild type and *Δrsp11* mutants. Wild type: (A-C), *Δrsp11* (D-F). **A.** Low power image through a microgametocyte showing the central nucleus (N) with a nuclear pole (NP) and the cytoplasm containing a number of axonemes (A). **B.** Detail from the cytoplasm of a mid-stage microgametocyte showing axonemes (A) with the normal 9+2 organisation. **C.** Detail of a cross section free flagellum (F) with a 9+2 axoneme. **D.** Low power of mutant microgametocyte showing the large central nucleus (N) with a nuclear pole (NP) with a number of axonemes (A) in the cytoplasm. **E.** Detail from the cytoplasm showing two cross-section axonemes with the normal 9+2 organisation. **F.** Cross section through a microgamete showing the nucleus (N) and 9+2 axoneme (A). Bars represent 1 um (A and D) and 100 nm (B, C and E, F).

Journal of Cell Science • Supplementary information

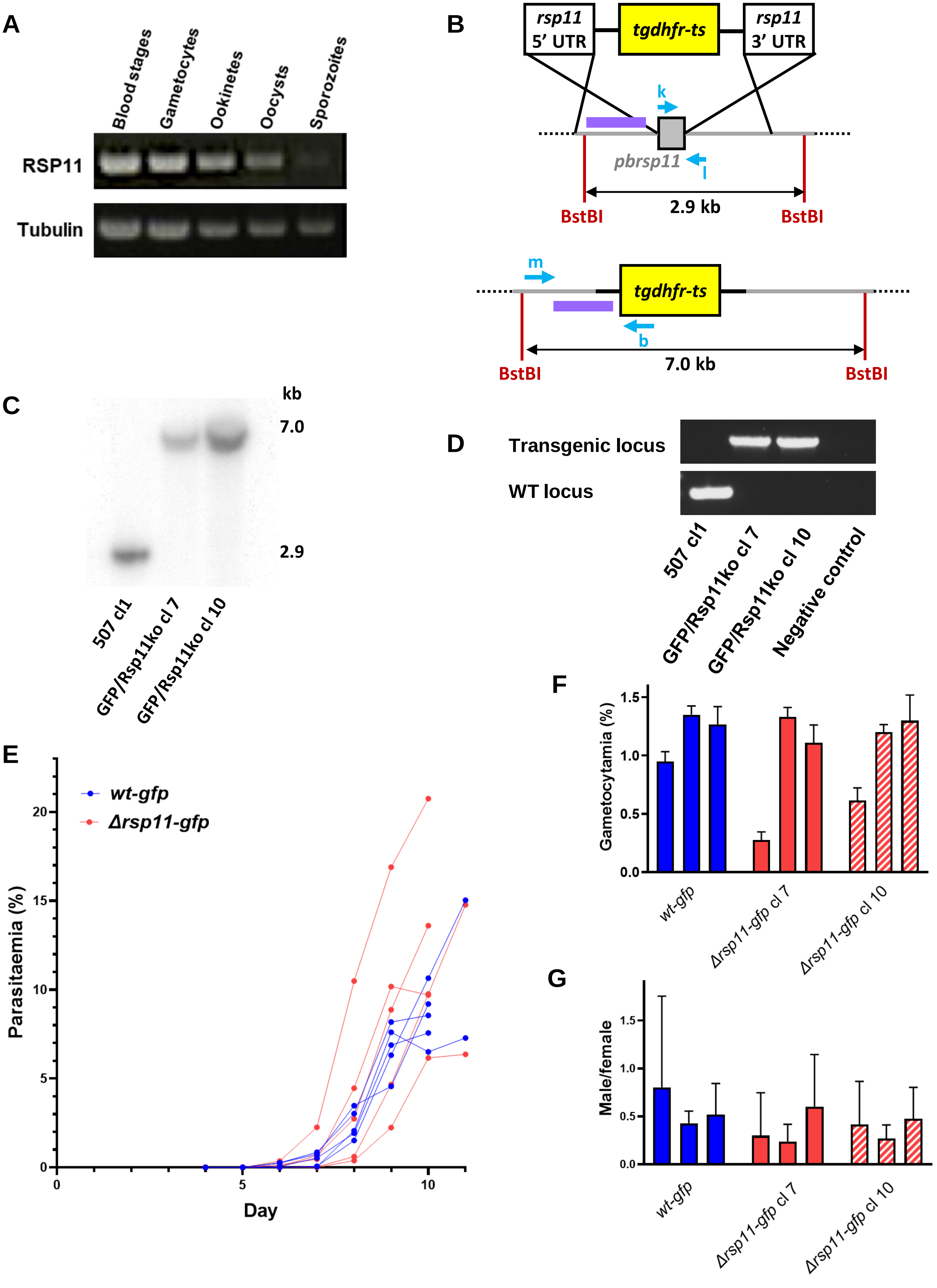

Journal of Cell Science • Supplementary information

H

	15 min		20 min	
	Exflagellating gametocytes (%)	Released flagella/ exflagellating gametocyte (%)	Exflagellating gametocytes (%)	Released flagella/ exflagellating gametocyte (%)
wt-gfp	54.51	11.72	66.23	24.50
***Δrsp11-gfp* cl 7**	41.64	1.52	48.52	4.35

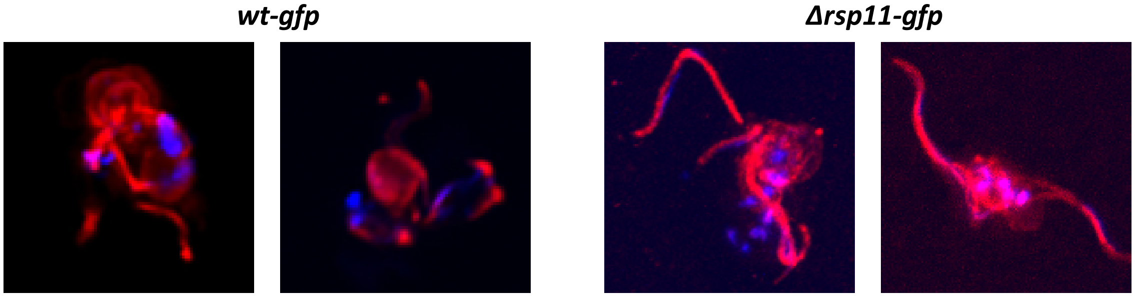

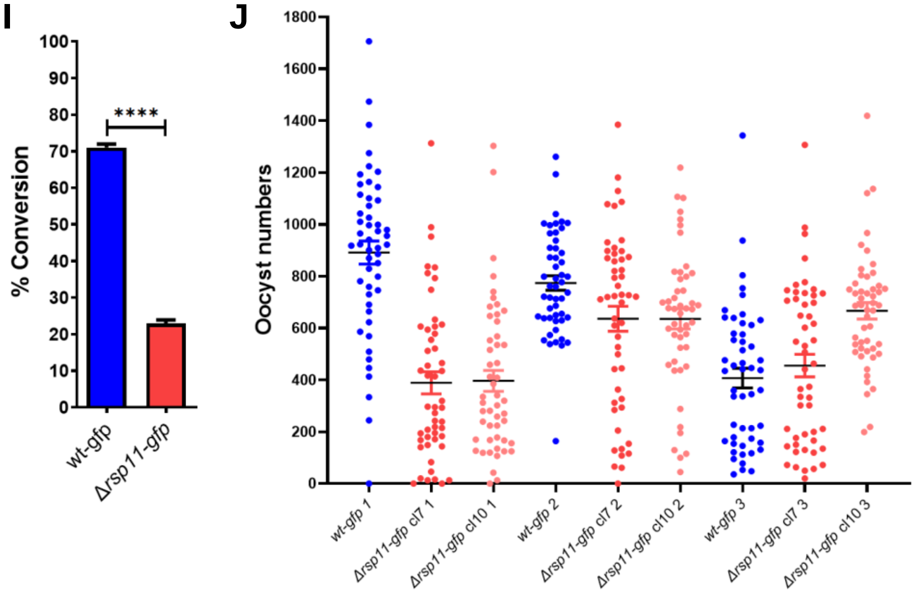

K

Strain	Mice infected
Δrsp11-gfp cl7	4/4
Δrsp11-gfp cl10	4/4
wt-gfp	4/4

Fig. S4. PBANKA_112580 predicted to be RSP11 is not a *Plasmodium* flagellar protein

A. *Pbrsp11* RT-PCR analysis of mixed blood stages, gametocytes, ookinetes, oocysts and sporozoites. PCR using the constitutively expressed *tubulin* gene served as control for RNA/cDNA quality. **B.** Homologous recombination of the *Toxoplasma gondii* dihydrofolate reductase thymidylate synthase (*Tgdhfr-ts*) gene into the *P. berghei rsp9* locus resulting in gene deletion. The Southern blot probe is illustrated as purple bar. **C.** Analysis of mutant locus in transgenic lines after plasmid integration by Southern blot. Restriction digestion of genomic DNA with BstBI produces fragments of different sizes for *Δrsp9-gfp* (7 kb) and *wt-gfp* (2.9kb). The Southern probe was generated by amplification of the 5′ UTR region. **D.** Diagnostic PCRs of *Δrsp9-gfp* and *wt-gfp* parasites. Primer positions are illustrated in figures **B. E**. Asexual growth of *Δrsp11-gfp* clones and *wt-gfp* recorded from onset of parasitaemia. **F**. Gametocytaemia and (**G**) gametocyte sex ratio were determined by infections of 3 mice each infected with *Δrsp11-gfp* or *wt-gfp*. Error bars represent standard errors of the means. **H.** Assessment of exflagellation and gamete release rate of *Δrsp11-gfp* compared to *wt-gfp*. Anti-α-tubulin staining of exflagellating *wt-gfp* and *Δrsp11-gfp* male gametocytes illustrates exflagellation processes similar to wildtype in *Δrsp11-gfp.* Anti-tubulin is visualized in red with DAPI as nuclear counter stain. **I**. Ookinete conversion rate of *Δrsp11-gfp* clones and *wt-gfp*. Error bars represent standard error of the mean (SEM). Asterisks represent a p-value < 0.0001 determined by Welch's t-test. **J**. Number of oocysts *Δrsp11-gfp* clones in mosquito midguts compared to *wt-gfp*. Bars depict mean with SEM. **K.** Infection of C57Bl/6 mice with mosquitoes infected with *Δrsp11-gfp* clones and *wt-gfp* result in blood stage parasitaemia in all mice at day 6 post infection.

Journal of Cell Science • Supplementary information

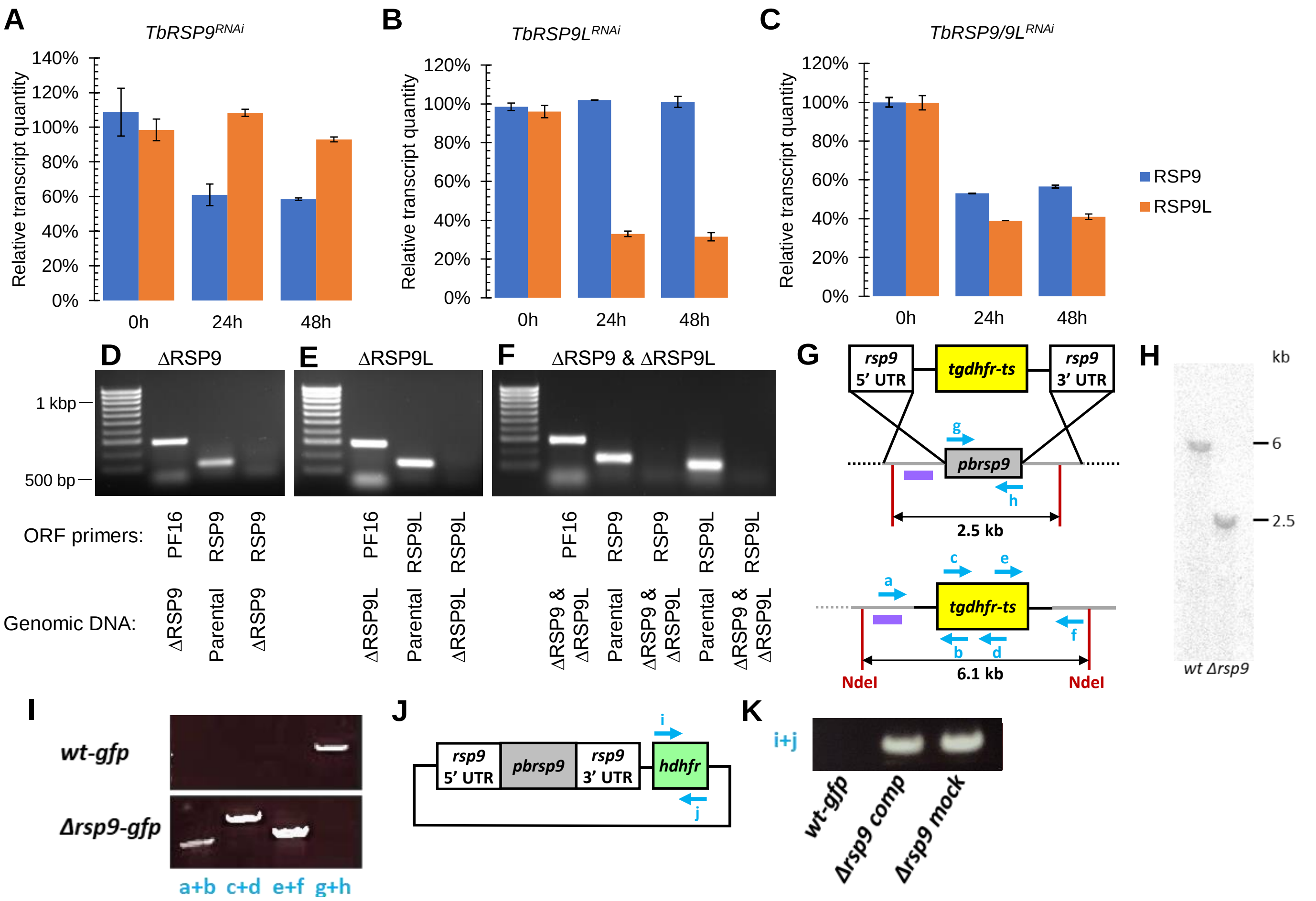

Fig. S5. Validation of RSP9 mutants

A-C. Diagnostic qRT-PCR to determine the knockdown of TbRSP9 and TbRSP9L in the **A**. TbRSP9^{RNAi} , **B.** TbRSP9L^{RNAi} and **C.** TbRSP9 & RSP9L^{RNAi} *T. brucei* cell lines. For each, the relative transcript quantity at 0, 24 and 48 h following induction are shown. Error bars represent the standard deviation of *n* = 3 technical replicates. **D-F.** Diagnostic PCRs to confirm deletion of both alleles of **D**. LmxRSP9, **E**. LmxRSP9-Like and **F**. both LmxRSP9 and LmxRSP9-like in the clonal *L. mexicana* deletion cell lines. For each, gel electrophoresis of PCR products from genomic DNA (gDNA) are shown. Control PCR products from an unaffected open reading frame (ORF), PF16, are shown to confirm presence of deletion mutant gDNA and PCR products from parental gDNA are shown to confirm that the test primers can amplify the RSP9/RSP9-like ORF. **G.** Homologous recombination of the *Toxoplasma gondii* dihydrofolate reductase thymidylate synthase (*Tgdhfr-ts*) gene into the *P. berghei rsp9* locus resulting in gene deletion. Arrows represent primers used for diagnostic PCR; the Southern blot probe is illustrated as purple bar. **H.** Analysis of mutant locus in transgenic lines after plasmid integration by Southern blot. Restriction digestion of genomic DNA with NdeI produces fragments of different sizes for *Δrsp9-gfp* (6 kb) and *wt-gfp* (2.5 kb). The Southern probe was generated by amplification of the 5′ UTR region. **I.** Diagnostic PCRs of *Δrsp9-gfp* and *wt-gfp* parasites. Primer positions are illustrated in figures **G. J.** Scheme of the plasmid used to transiently express *rsp9* to complement the knock-out phenotype. The *hdfhr* gene confers resistant to WR99210. Arrows illustrate primers used for diagnostic PCRs. **K.** Diagnostic PCRs of *Δrsp9-gfp*-rescue parasites compared to the mock transfected *Δrsp9-gfp.* Primer positions are illustrated in panel J.

Journal of Cell Science • Supplementary information

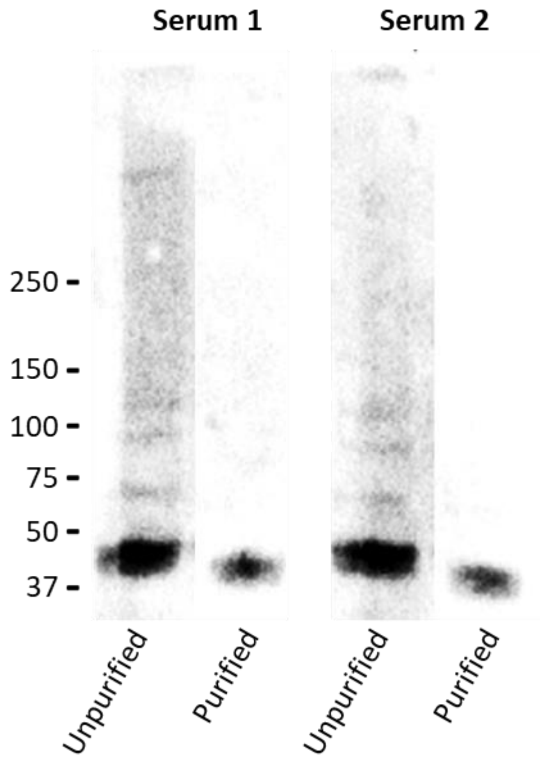

Fig. S6. Validation of PfRSP9 anti-sera

Western blot analysis of activated *P.falciparum* mixed gametocytes separated by native PAGE. Sera and affinity-purified immunoglobulins using coupled peptides have been tested and shown to recognize a single band at 43 kDa corresponding to the expected molecular weight.

Table S1. Orthologs of *Chlamydomonas reinhardtii* RSPs in *P. falciparum*, *P. berghei*, *T. brucei* and *L. mexicana*

Click here to download Table S1

Table S2. Gene IDs and primer sequences

Click here to download Table S2

Table S3. Amplicon length, qPCR efficiencies and r^2 of calibration curve for *T. brucei* RSP9

Gene	Description	Amplicon length	qPCR Efficiency	r^2
Actin	Actin A	147bp	97.4%	0.9994
Aldo	Fructose-bisphosphate aldolase glycosomal	86bp	97.8%	0.9997
MRE11	DNA Repair protein	111bp	100.8%	0.9981
ODA7	Outer row dynein-assembly protein 7	118bp	100.7%	0.9993
RSP9	Radial spoke protein RSP9	85bp	93.7%	0.9980
RSP9-L	Radial spoke protein RSP9-Like	90bp	96.3%	0.9979
TERT	Telomerase reverse transcriptase	109bp	92.4%	0.9971

Table S4. Prevalence and number of mosquitoes, average oocysts per mosquitoes and number of mosquitoes observed

Strain	Experiment	Mosquitoes fed	Mouse infected	Sporozoite load per mosquito (n)
Δrsp9-gfp	1	33	Yes	1736 (53)
Δrsp9-gfp	2	19	Yes	17 (30)
Δrsp9-gfp	3	31	Yes	29'575 (31)
wt-gfp	4	5	Yes	911 (10)
wt-gfp	5	6	Yes	206 (10)
wt-gfp	6	5	Yes	25'575 (8)

Table S5. Number of *Δrsp9-gfp* oocysts in mosquito midguts compared to infections with *wt-gfp*.

Strain	Experiment	Number of mosquitoes	Average number of oocysts	Prevalence (%)
Δrsp9-gfp	1	49	38	79
Δrsp9-gfp	2	35	13	44
Δrsp9-gfp	3	63	32	35
wt-gfp	1	50	582	92
wt-gfp	2	50	748	95
wt-gfp	3	26	679	72
