# Supplementary material for: Radial spoke protein 9 is necessary for axoneme assembly in Plasmodium but not in trypanosomatid parasites
Source: J Cell Sci. 2023 Jun 8;136(11):jcs260655. doi: 10.1242/jcs.260655 (PMC10309588; doi:10.1242/jcs.260655)
Supplement: Supplementary file 1 [file joces-136-260655_review_history.pdf]

Radial spoke protein 9 is necessary for axoneme assembly in *Plasmodium* but not in trypanosomatid parasites

Chandra Ramakrishnan, Cécile Fort, Sara Rute Marques, David J. P. Ferguson, Marion Gransagne, Jake Baum, Soraya Chaouch, Elisabeth Mouray, Linda Kohl, Richard J. P. Wheeler and Robert E. Sinden
DOI: 10.1242/jcs.260655

Editor: Jennifer Lippincott-Schwartz

Review timeline

Original submission:	22 September 2022
Editorial decision:	18 November 2022
First revision received:	7 January 2023
Editorial decision:	10 February 2023
Second revision received:	19 April 2023
Accepted:	9 May 2023

Original submission

First decision letter

MS ID#: JOCES/2022/260655

MS TITLE: Radial spoke protein 9 is necessary for axoneme assembly in Plasmodium but not in trypanosomatid parasites

AUTHORS: Chandra Ramakrishnan, Cecile Fort, Sara Rute Marques, David J. P. Ferguson, Marion Gransagne, Jake Baum, Soraya Chaouch, Elisabeth Mouray, Linda Kohl, Richard John Wheeler, and Robert E. Sinden
ARTICLE TYPE: Research Article

We have now reached a decision on the above manuscript.

To see the reviewers' reports and a copy of this decision letter, please go to: https://submit-jcs.biologists.org and click on the 'Manuscripts with Decisions' queue in the Author Area. (Corresponding author only has access to reviews.)

As you will see, the reviewers raise a number of substantial criticisms that prevent me from accepting the paper at this stage. They suggest, however, that a revised version might prove acceptable, if you can address their concerns. If you think that you can deal satisfactorily with the criticisms on revision, I would be pleased to see a revised manuscript. We would then return it to the reviewers.

Please ensure that you clearly highlight all changes made in the revised manuscript. Please avoid using 'Tracked changes' in Word files as these are lost in PDF conversion.

I should be grateful if you would also provide a point-by-point response detailing how you have dealt with the points raised by the reviewers in the 'Response to Reviewers' box. Please attend to all of the reviewers' comments. If you do not agree with any of their criticisms or suggestions please explain clearly why this is so.

© 2023. Published by The Company of Biologists under the terms of the Creative Commons Attribution License (https://creativecommons.org/licenses/by/4.0/).

Reviewer 1

Advance summary and potential significance to field

By focusing on the radial spoke of the axoneme of Plasmodium berghei, this manuscript uncovers the strategy that evolved in the organism for stripping down an exceedingly complex structure into a still functional organelle. The experiments are well-done, the results are compelling, and the findings will be of interest across the broad readership of the Journal of Cell Science.

Comments for the author

Below, I list several, more detailed comments, which can also been seen in the annotated pdf file of the manuscript.

Line 48: I think it is difficult to ask or answer "why" questions in biology. Consider discussing function.

Line 62: Not sure that results reveal much about evolution? They do provide insights into the multiple ways that the organelle can been constituted to carry out organism-specific functions.

Line 70: Cell biologists who don't work on parasites are not likely to know what a transmissive metacyclic stage cell is. Need to provide more information and to describe the importance of motility for this type of cell.

Line 72-73 (“axoneme growth and flagellar formation occur in parallel”): Since the axoneme forms the core of the flagellum, readers might not understand the reason for making this statement, because it seems to go without saying. Of course, the sentence makes sense once flagellum formation in the two organisms is described.

Line 75: Replace “These IFT ‘trains’” with “IFT particles enter the flagellum in linear arrays called trains that.”

Line 83: Need to clarify this sentence. Is a gametocyte a Plasmodium spp? cell, or is a gametocyte a red blood cell with a Plasmodium spp? cell within it?

Line 94: The citations should be re-considered here, since IFT was not known in 1976.

Line 106a: Consider adding EMs here, or in supplement, showing radial spokes in the flagella of these cells.
Even though images of radial spokes in wt cells are shown later, it might help the reader to see them at this place in the manuscript.

Line 106b: Use of term "thus?" Not necessarily obvious that the preceding information naturally leads to the conclusion that it is important to study radial spoke proteins?

Line 110: Replace “this illustrates” with “we found.”

Line 118: Need to describe an RS "head."

Line 121: Meaning unclear? Otherwise dispensable for what?

Line 134: Fig. 1A shows information about the presence of RSP protein family members in organisms across multiple taxa, but the manuscript does not discuss the findings. For example, given that Arabidopsis lacks cells with flagella, the authors should comment on the presence of RSP protein in this and related organisms. If the authors feel that such a discussion is out of the scope of this manuscript, then the findings for the organisms or groups of organisms that are out of the scope should be omitted from the main text. Possibly, they could be included as supplemental information, but again, unless the results are discussed, probably they don't belong in the manuscript. I should note that this type of information truly does address the evolution of RSP

© 2023. Published by The Company of Biologists under the terms of the Creative Commons Attribution License (https://creativecommons.org/licenses/by/4.0/).

proteins, and the authors might consider making some brief comments about the evolutionary distributions and provide speculative interpretations.

Line 148: Need a negative control sample for the TrypTag samples to confirm that indeed not every tagged protein localizes to the flagellum.

Line 150+: Using the genus name Plasmodium without indicating the species name is problematic. If I understand Fig. 1A and its legend correctly, Pf has a single ortholog for RSP9, whereas Pb has multiple RSP9 orthologs. Yet line 150 and 151 say that Plasmodium (with no species name) possesses only a single ortholog?

Figure 1A: The use of an open circle to designate the presence of a single ortholog is confusing. At first without reading the legend, I assumed that an open circle meant the absence of an ortholog. Possibly, I should have immediately understood the figure, and possibly this method of presentation is conventional but I predict that I would not be alone in being confused. I suggest that authors provide a different, less ambiguous designation.

Line 159: In Figure 1B legend, need to provide much more information about the models. For example, does the Pb model depict the appearance of a Chlamydomonas radial spoke containing only the Chlamydomonas version of RSP4/6 and RSP9? Or, are the predicted structures of Pb RSP4/6 and RSP9 shown? It is instructive to present such models, but it was hard to understand what was being modeled? (Also, see below.)

Line 161: Fig. 1C labels need to be adjusted to avoid being masked by portions of the predicted structures.

Line 162: Text needs to provide more detail about the differences in complexity among the 3 RS1s illustrated in the cartoons. Given that Plasmodium lacks several RSP proteins, it goes without saying that the Plasmodium structure would be simpler. It would be appropriate here and, in the Discussion, to highlight which parts of the structures are different, to speculate on whether the differences would be reflected in the overall structure, and to speculate on possible functional consequences. For example, it seems to make sense that the top-most part of the RS1 complex, i. e., RSP9, would be the one that is retained in a stripped-down version, but the text needs to describe and explain this idea more fully.

Line 173, Figure 1C. It would be helpful to provide the structures of the acidic loops of Chlamydomonas RSP4 and 6. The authors should elaborate about the significance of the similarities of the acidic loops of Pb RSP9 and the RSP4 and RSP6 proteins.

Line 265/Fig. 4F: The data in Fig. 4F are central to support a major finding of this manuscript, yet the images are not compelling. Both wt and the rsp9 mutant gametocytes exhibit substantial anti-tubulin staining (lots of red stuff, some possibly linear?). The images should be larger and the text needs to do a better job of describing and labeling the flagella for a reader not familiar with viewing Plasmodium spp? exflagellating male gametoctytes.

Line 305: Figure 6 legend: should be E-I not E-!.

Line 306: Figure 6 legend: I would disagree with the descriptions of microtubules in the mutant gametocyte. In my view, the 6 doublet (not "duplet") microtubules in F are in a linear array and thus have a non-random relationship to each other. I would guess that they represent 6 doublets that are linked laterally as if they started out as part of a set of 9 outer doublets that failed to remain together during attempted assembly. In addition, because of their close relationship to each other, my guess is that the 2 singlet microtubules in 6F did not just happen to form near each other, but likely represent a central pair that failed to become incorporated into an intact axoneme. The authors might not agree with my interpretation of these images, but I think my interpretations are within the realm of possibilities and at least should be presented, along with reasons for discounting the idea.

Along these lines, it would be helpful if the authors reminded the reader about current models of axoneme assembly and which step might require RSP9. I expect it has been shown the Plasmodium

© 2023. Published by The Company of Biologists under the terms of the Creative Commons Attribution License (https://creativecommons.org/licenses/by/4.0/).

spp? basal body has triplet microtubules, and that the A and B tubules of each triplet provide that template for assembly of the outer doublet microtubules. For example, based on these results, my prediction would be that the doublets are nucleated in the mutant, that they form side-to-side associations as they extend, but that they fail to stay together as a 9-part unit because RSP9 provides an additional mechanism for connecting them?
Thinking along these lines also brings to mind the question of whether or not dynein-driven microtubule sliding might force the doublets apart? Although not at all a requirement for this manuscript, in future experiments it would be interesting to learn whether intact axonemes would form in RSP9 mutants if they also lacked a dynein arm essential for motility?

<u>Reviewer 2</u>

Advance summary and potential significance to field

Summary: Systematic analysis of RSP9 and RSP9L in T. brucei, Leishmania, and RSP9 in Plasmodium berghei. The authors present a thorough analysis of RSP9/9L representation in various eukaryotes, as well as fluorescence localization data demonstrating flagellum location for the proteins in each organism. They then use knockdown (T brucei) or knockout (Leishmania and Plasmodium) to assess function. T brucei and Leishmania show motility defects in absence of either protein without growth rate deficit. Leishmania shows clear imact on radial spoke heads in each mutant, while the case isn't entirely clear for T. brucei owing to low efficacy of knockdown. Plasmodium shows defects in motility and exflagellation by male microgametes, as well as profound, axoneme architecture defects beyond simply radial spoke defects.

Overall the work is sound, with ample data to support most conclusions. It is well-written and should be of interest to a broad audience, particularly the much-needed functional analysis of flagellar proteins in Plasmodium. I have a few comments to share for improving the manuscript.

Comments for the author

• The only major concern I have relates to conclusions about protein ‘requirement’ in T. brucei. For example, in the Abstract, lines 29-30: the statement that RSP9 orthologs are not necessary for axoneme assembly should be adjusted. The extent of knockdown (~40% in one case and ~70% in another) means that a substantial amount of mRNA remains present, and this level of expression could accommodate axoneme assembly. The authors are good at addressing this in the main text, e.g. line 205, but the statement in the abstract does not express this important caveat regarding presence of axonemes in RSP9/9L knockdowns.

Results in Leishmania, with complete KO, supports the general view, but care should still be taken in making the claim for T. brucei. A similar concern/caveat holds for lines 294-296. The statement says RSP9, 9L are "'both' necessary for RS head assembly...". Data indicate they are 'each' necessary in Leishmania, RSP9L is required in T. brucei, and the result is equivocal for RSP9 in T. brucei because no clear defect was observed, yet knockown efficacy was marginal. The statement needs to be re-worded accordingly.

• line 58: Grossman-Haham seems appropriate to also cite here.
• lines 65-66. It should be noted that motility is also required for progression through the insect vector and pathogenesis in the mammalian host
(rotureau 2014b; shimogawa 2018 that are cited here). To a general audience, that is likely not clear from simply saying "life cycle".
• line 104: is it accurate to say plasmodium display "canonical" 9+2 axonemes? In the EM images presented (Fig 6 and S3) it isn't clear if they have canonical spokes. Even the fuzzy electron dense material seen between outer doublets and central pair appears to extend more from the dyneins, rather than the doublet. Having only 3 of 23 or more canonical radial spoke proteins, it is hard to imagine plasmodium having canonical spokes. it's perhaps a nuance, but would be worth adjusting this comment, and this could be very informative and enlightening for the community, as a solid description of the wt axoneme would be of interest.

© 2023. Published by The Company of Biologists under the terms of the Creative Commons Attribution License (https://creativecommons.org/licenses/by/4.0/).

- line 170: should read "...and that TbRSP9 and PbRSP9 PREDICTED structures are very similar to..."
- Line 348: is it clear that axoneme assembly in Plasmodium is "error prone"? some images are shown of axonemes without a central pair, but it is unclear if these represent abnormal flagella after complete assembly, vs a portion of an axoneme that is yet incomplete, or some other axoneme that is in the range of 'normal assembly processes.
- Methods 5.10. please include the reference for motility sedimentation assay in the methods section. The section itself is quite brief, understandably if this is a standard assay, but a reference for a more complete description should be provided.
- Line 571: For ookinete formation, it would be helpful to see representative images of the distinguishing features used to quantitate ookinetes.
- RESULTS, subheading 3.5.
- The heading should be adjusted to reflect the finding that spoke heads are affected. The heading simply states ...not essential for axoneme structure...
- The mixed cross using defined female-defective or male-defective plasmodium mutants is clever and informative
- What explains the lowered parasitemia in late infection timepoints for the RSP9 KO, considering gametocytogenesis is unaffected.
- Fig 1: It would be informative to include the alpha fold prediction for the Chlamydomonas protein, to see how this compares to the experimentally determined structure.
- Fig 4: The legend appears to have a typo, with panel F labeled as panel

'b. Fig 4, panel F is problematic: There is not sufficient clarity to discern differences between the wt and rsp9-KO. one panel of the KO looks unlike the other three panels, but the other three panels look, for most purposes, to be similar. the authors need to provide a) examples of whole fields of samples and some quantitative analysis, to evaluate how representative the stated defect is; and b) a descriptive panel in which the relative features that are important are clearly labeled. The result is a central finding of the work and needs some more demonstrated support.

<u>Reviewer 3</u>

Advance summary and potential significance to field

The authors investigated heterogeneity in radial spoke proteins (RSPs) across several species and then performed an investigation into the role of the orthologs of RSP9 in Plasmodium and trypanosomatids. They verified RSP9 ortholog localization to flagella and discovered that loss of the RSP9 ortholog had differential effects on flagella depending on the organism. In T. brucei and L. Mexicana flagella formed however swimming was impaired. In P. berghei flagellar assembly was disabled. The authors conclude that the different requirements for RSP9 are due to different selection pressures related to the mechanism of flagellar assembly.

As written the manuscript does not meet the Journal standard for making a significant and novel contribution to our understanding of cell biology of broad interest. However, I am optimistic that a revised manuscript could meet the standard. I have provided details below, but in general, the motivation and significance of the research are under-developed and the bioinformatic and experimental studies are insufficiently integrated. In addition, there are technical concerns with the data presented that I hope can be easily addressed.

Comments for the author

Summary of the paper:
The authors investigated heterogeneity in radial spoke proteins (RSPs) across several species and then performed an investigation into the role of the orthologs of RSP9 in Plasmodium and trypanosomatids. They verified RSP9 ortholog localization to flagella and discovered that loss of the RSP9 ortholog had differential effects on flagella depending on the organism. In T. brucei and L. Mexicana flagella formed however swimming was impaired. In P. berghei flagellar assembly was disabled. The authors conclude that the different requirements for RSP9 are due to different selection pressures related to the mechanism of flagellar assembly.

© 2023. Published by The Company of Biologists under the terms of the Creative Commons Attribution License (https://creativecommons.org/licenses/by/4.0/).

As written the manuscript does not meet the Journal standard for making a significant and novel contribution to our understanding of cell biology of broad interest. However, I am optimistic that a revised manuscript could meet the standard. I have provided details below, but in general, the motivation and significance of the research are under-developed and the bioinformatic and experimental studies are insufficiently integrated. In addition, there are technical concerns with the data presented that I hope can be easily addressed.

Motivation and significance:
• The motivation listed in the abstract is “flagella are important for motility”. Because you are studying parasites that require flagella for their life cycle, and investigating difference, a more extensive motivation could be presented.
• To more clearly communicate the value of the work you have done, define the unanswered question that you answer in this study (include it in the abstract, introduction and answer it in the discussion). In the Intro you write: “it is not known precisely why RSs are important…” This study does not answer that question.
• Establish a motivation for examining functional conservation of RSP9 after identifying all RS in so many species.

Integration of bioinformatic and experimental analysis:
In the current draft, the bioinformatic analysis competes with the analysis of RSP9 instead of motivating or complimenting it. Here are a few specific points, but the integration may require more than just addressing these.
• Why is a comprehensive analysis of diverse organisms useful to identify the orthologs in 4 species to the RSPs in Chlamydomonas?
• Results from the bioinformatic analysis are detailed both in the second to last paragraph of the introduction as well as the first 3 paragraphs of the Results section
• 4 of the 5 supplemental figures are not about RSP9

One possible strategy for you to consider:
There are multiple ways that one could more clearly communicate the motivation and significance of the study and to integrate the different approaches. Here is one possibility that occurred to me.
• All motile cilia and flagella have RS, but they are not the same in all species. In species examined to date there is variability between the component proteins

• Loss/mutation of RSPs causes defects in conventional model organisms suggesting mechanical/mechanosensitive function. It is possible that the RS has the potential to serves multiple functions within a single flagellum
• Unanswered question: How do RSPs contribute differently to flagellar assembly and function in parasite species that depend on flagella for life cycle?
• Use bioinformatics to identify an RSP that can be used to explore this conundrum
• Identify RSP9

Important issues with the data that need to be addressed:
• Two Plasmodium species are used in this study. It is unclear which is examined in some figures and why each was analyzed where it was. In figure 1B, C, & D, Plasmodium berghei is featured. In figure 2 there are images of Plasmodium falciparum. I think Figure 4 and 6 feature P. berghei because line 231 states that the knock-out lines are made in that organism, however this is not indicated anywhere else in the results, figure legend or figure.
• The staining in Figure 2D is not convincing. What is the intense central signal that is not similar to the alpha tubulin. Include data or a reference to support that the PfRSP9 antisera is specific for RSP9. Also include additional example images.
• Figure 3 A and C should include data from replicates with statistical analysis instead of representative plots.
• The conclusions drawn from the T. brucei knock downs (including lines 218 & 219 and the discussion) should be moderated to account for the partial knockdown. One cannot conclude that removal of the protein has no affect on assembly when the protein has not been completely removed.
• Figure 4A requires additional statistical analysis. The displayed variance makes it difficult

© 2023. Published by The Company of Biologists under the terms of the Creative Commons Attribution License (https://creativecommons.org/licenses/by/4.0/).

to have confidence in the conclusions drawn from the data. In the manuscript day 9 is highlighted for comparison, however no mention is made that some trials do not extend as far as day 9. Perhaps additional trials will be necessary to discern if statistically robust differences exist.
• The description of the experiment presented in Figure 4E was insufficient for me to evaluate the data. Additional background about the mutant cell lines and motivation for the experiment could hopefully resolve this easily.
• The EM presented in Figure 6 is essential and should be expanded.
o Fig. 6E in the legend is described as a section showing the absence of axonemes.
How is this consistent with the IF shown in Figure 4 where the flagella are described as "either wrapped around the gametocyte body or protruding only partially"?
Have all of the sections been assessed?
Can you make more connections between how the EM explains what you see by IF?
o Are basal bodies present or disrupted?
• In line 319 (Discussion) the statement that "the overall structure of RSs, as visualized by electron microscopy, is very well conserved across all ciliated/flagellated organisms…" seems contradicted by visual differences in RS in Figure 5.
o (I really like Figure 5, by the way. It is presented and described clearly and the differences are interesting!)

Minor Points:
• Include catalog numbers in the methods section – especially for antibodies.
• In every figure legend indicate the organisms that have been used. For example, in Figure 3, T. brucei and L. Mexicana are only identified in the figure title and should be included to indicate that A, B and C are T. brucei and D-G are L. Mexicana.
• Line 770: "(E-!)" should be "(E-I)"

First revision

Author response to reviewers' comments

We would like to thank you for considering a revised version of manuscript JOCES/2022/260655. We are grateful to all three reviewers for their constructive criticisms and appreciate their feedback to make this a better manuscript. Below, we have addressed the issues point by point and have also amended the manuscript accordingly.
The major amendments are:
- Correction and adjustments of figures and their legends, notably Figure 1A.
- Modified/added text for clarity.
- Modified the abstract to take into account reviewers' comments.
- Amended mistakes.
- Added catalogue numbers for antibodies.

We have documented all changes in our rebuttal below and in the main text, changes are highlighted in yellow and removed text is crossed out.

We thank you and the reviewers for considering this improved version of the manuscript for publication in the Journal of Cell Sciences.

We are looking forward to your reply,

Reviewer 1

Line 48: I think it is difficult to ask or answer "why" questions in biology. Consider discussing function.
This is not a stand-alone 'why', it was intended as a run-in to the detailed mechanistic/functional description of the second half of a sentence. We have rephrased this. (line 49).

© 2023. Published by The Company of Biologists under the terms of the Creative Commons Attribution License (https://creativecommons.org/licenses/by/4.0/).

Line 62: Not sure that results reveal much about evolution? They do provide insights into the multiple ways that the organelle can been constituted to carry out organism-specific functions.
The fact that multiple functional constitutions of RSPs in diverse organisms have arisen is an evolutionary process. We have rephrased the sentence to indicate that the differing assembly mechanisms may exert a different selection pressure on assembly.

Line 70: Cell biologists who don't work on parasites are not likely to know what a transmissive metacyclic stage cell is. Need to provide more information and to describe the importance of motility for this type of cell.
We have added a more detailed description to expand on the meaning of a transmissive stage for a parasite. Note that it is unknown what the importance of this life cycle stage is – the text as written, that the cell is highly motile, is correct. The functional consequences of this remain somewhat cryptic. (line 70)

Lines 72-73: “axoneme growth and flagellar formation occur in parallel”: Since the axoneme forms the core of the flagellum, readers might not understand the reason for making this statement, because it seems to go without saying. Of course, the sentence makes sense once flagellum formation in the two organisms is described.
We have rephrased this to try to clarify – we agree that the term “parallel” was potentially confusing as it implies a spatial parallel organisation. Note that, for general cell biologists, we emphasise in the first half of the sentence that this is canonical assembly. (line 83)

Line 75: Replace “These IFT ‘trains’” with “IFT particles enter the flagellum in linear arrays called trains that.”
This sentence has been modified to “IFT particles enter the flagellum in linear arrays called “trains” that move along the microtubules using motor proteins powered by ATP.” . (line 86)

Line 83: Need to clarify this sentence. Is a gametocyte a Plasmodium spp? cell, or is a gametocyte a red blood cell with a Plasmodium spp? cell within it?
We appreciate the reviewer’s comment and the fact that JCS is not a specialised parasitology journal. Gametocytes in all apicomplexan parasites are a term for sexual parasite stages that produce gametes. In Plasmodium spp., the gametocytes reside in red blood cells. We have now changed the text to: “Gametocytes are (red blood cells containing pre-gamete parasites intraerythrocytic male and female sexual stages that are quiescent in the vertebrate host”. (line 96)

Line 94: The citations should be re-considered here, since IFT was not known in 1976.
This citation was slightly misplaced. The 1976 citation refers to evidence for a non-canonical assembly, and we have added a new citation for the IFT independence: Briggs et al.,Curr Biol. 2004 14(15):R611-2. doi: 10.1016/j.cub.2004.07.041 . (line 108)

Line 106a: Consider adding EMs here, or in supplement, showing radial spokes in the flagella of these cells. Even though images of radial spokes in wt cells are shown later, it might help the reader to see them at this place in the manuscript.
We understand the reviewer’s point, but would like to point out that the listed references provide appropriate images of WT axonemes. We believe that adding our own images as another figure in the introductory part of the paper would not add much value, but would disrupt the order of the figures. Because we have clearly illustrated the appearance of the wild-type radial spokes in Figure 6 we have left this unchanged.

Line 106b: Use of term "thus?" Not necessarily obvious that the preceding information naturally leads to the conclusion that it is important to study radial spoke proteins?
We have rephrased this to try to clarify the logic. “As there may be differences in RSs associated with these differing mechanisms of flagellum assembly we aimed to study radial spoke proteins that are essential for flagellar beating in these parasites.” (line 125)

Line 110: Replace “this illustrates” with “we found.”
This sentence has been modified to “We found that, similar to the reference organism (Chlamydomonas), many organisms, including trypanosomatids, possess a large number of RSPs” . (line 128)

© 2023. Published by The Company of Biologists under the terms of the Creative Commons Attribution License (https://creativecommons.org/licenses/by/4.0/).

Line 118: Need to describe an RS "head."
We described the shape of the RS earlier and have added a short description of the RS head (line 54, new version) “and the enlarged “head” projecting towards the central pair of the axoneme”. We have also clarified the position of RSP9 in the “head” portion of the RS “the RS protein RSP9, which forms part of the enlarged ‘head’ of the RSP mushroom shape”. (line 134)

Line 121: Meaning unclear? Otherwise dispensable for what?
To take also into account a comment from reviewer 2, this has been modified to “Using gene deletion in L. mexicana, we show both RSP9 and RSP9-like are necessary for RS head assembly and normal cell motility but are not needed for cell survival. Similar results were obtained by RNAi knockdown in T. brucei.” (line 137)

Line 134: Fig. 1A shows information about the presence of RSP protein family members in organisms across multiple taxa, but the manuscript does not discuss the findings. For example, given that Arabidopsis lacks cells with flagella, the authors should comment on the presence of RSP protein in this and related organisms. If the authors feel that such a discussion is out of the scope of this manuscript, then the findings for the organisms or groups of organisms that are out of the scope should be omitted from the main text. Possibly, they could be included as supplemental information, but again, unless the results are discussed, probably they don't belong in the manuscript. I should note that this type of information truly does address the evolution of RSP proteins, and the authors might consider making some brief comments about the evolutionary distributions and provide speculative interpretations.
The critical value of showing these other species is as a reference/control to show which detected RSP orthologs have a flagellar-specific function (only in flagellate species) and which do not – this has been clarified in the text. E.g. it shows that the Pf RSP9 orthogroup member is likely involved in the flagellum, but the Pf protein in the RSP16 orthogroup is not.

Some ‘outliers’ like RSP10 in Arabidopsis may be true orthologs, implying a non-flagellar gain of function, or false positives. As we do not perform a detailed analysis of these proteins, we do not draw a specific conclusion.

Line 148: Need a negative control sample for the TrypTag samples to confirm that indeed not every tagged protein localizes to the flagellum.
This is not primary data. The referenced genome-wide localisation project, from which these data are taken, showed that ~6% of proteins localised to the axoneme. Additional references demonstrating localisation to non-axoneme structures can be found at https://doi.org/10.1016/j.molbiopara.2018.12.003 or https://www.biorxiv.org/content/10.1101/2022.06.09.495287v1.

Line 150+: Using the genus name Plasmodium without indicating the species name is problematic. If I understand Fig. 1A and its legend correctly, Pf has a single ortholog for RSP9, whereas Pb has multiple RSP9 orthologs. Yet line 150 and 151 say that Plasmodium (with no species name) possesses only a single ortholog?
We apologise for the confusion; the legend had an error that we had missed. The precise meaning of the figure is that Pf has at least one RSP9 ortholog as defined by OrthoFinder orthogroup membership (there may be multiple, this is not shown) and Pb. has a reciprocal best BLAST hit (which is by definition singular). Both Pf and Pb have a single RSP9 ortholog.

Figure 1A: The use of an open circle to designate the presence of a single ortholog is confusing. At first, without reading the legend, I assumed that an open circle meant the absence of an ortholog. Possibly, I should have immediately understood the figure, and possibly this method of presentation is conventional, but I predict that I would not be alone in being confused. I suggest that authors provide a different, less ambiguous designation.
As described above, both interpretations are not correct because of our error in the figure legend. We have corrected the legend. We also updated the figure to a large vs. small point to represent a high confidence ortholog (RBB) vs a more sensitive but less precisely defined ortholog (OrthoFinder).

© 2023. Published by The Company of Biologists under the terms of the Creative Commons Attribution License (https://creativecommons.org/licenses/by/4.0/).

Line 159: In Figure 1B legend, need to provide much more information about the models. For example, does the Pb model depict the appearance of a Chlamydomonas radial spoke containing only the Chlamydomonas version of RSP4/6 and RSP9? Or, are the predicted structures of Pb RSP4/6 and RSP9 shown? It is instructive to present such models, but it was hard to understand what was being modeled? (Also, see below.)
We have clarified that these cartoons showing the C. reinhardtii structure, and then map ortholog presence/absence on this structure.

Line 161: Fig. 1C labels need to be adjusted to avoid being masked by portions of the predicted structures.
We have changed the species names to the abbreviated form so that they are smaller and no longer overlap.

Line 162: Text needs to provide more detail about the differences in complexity among the 3 RS1s illustrated in the cartoons. Given that Plasmodium lacks several RSP proteins, it goes without saying that the Plasmodium structure would be simpler. It would be appropriate here and, in the Discussion, to highlight which parts of the structures are different, to speculate on whether the differences would be reflected in the overall structure, and to speculate on possible functional consequences. For example, it seems to make
sense that the top-most part of the RS1 complex, i. e., RSP9, would be the one that is retained in a stripped-down version, but the text needs to describe and explain this idea more fully.
We have added text to clarify that this suggests that the minimal necessary components are RSP3 to position an RSP4/6-RSP9 tetramer by the central pair complex. (line 194)

Line 173, Figure 1C: It would be helpful to provide the structures of the acidic loops of Chlamydomonas RSP4 and 6. The authors should elaborate about the significance of the similarities of the acidic loops of Pb RSP9 and the RSP4 and RSP6 proteins.
We have added to the text that the RSP9 acidic loops may give rise to electrostatic interaction with the central pair complex analogous to that proposed to occur with the RSP4/6 acidic loops. (line 207)

It is not meaningful to include a predicted structure of the loops. These are likely unstructured loops, as can be predicted from the low complexity of their amino acid composition, failure to resolve the RSP9 and RSP4/6 loops in the cryoelectron microscopy data and the low pLDDT of these regions.

Line 265/Fig. 4F: The data in Fig. 4F are central to support a major finding of this manuscript, yet the images are not compelling. Both wt and the rsp9 mutant gametocytes exhibit substantial anti-tubulin staining (lots of red stuff, some possibly linear?). The images should be larger and the text needs to do a better job of describing and labeling the flagella for a reader not familiar with viewing Plasmodium spp? exflagellating male gametoctytes.
The anti-a-tubulin II antibody stains Plasmodium male gametocytes and gametes as the microtubule structure gets labelled. These seem intact in the mutant as well as shown by EM. To make it clearer to readers outside of the Plasmodium field, we have now added a more detailed explanation in the main text (line 291): “During this event, the male gametes beat vigorously to free themselves from the residual gametocyte body and finally being released. The free beating flagella are clearly visible as wavy threads protruding from the round gametocyte body.”

We have now also added to describe Fig. 4F better that if in mutant parasites, some protrusions can be visible, nuclei associated with the flagella are generally not observed (line 301). Additionally, we have added that the stain is microtubule-specific in the figure legend 4F and highlighted the nuclei with arrows.

Line 305: Figure 6 legend: should be E-I not E-!.
We have now corrected this.

Line 306: Figure 6 legend: I would disagree with the descriptions of microtubules in the mutant gametocyte. In my view, the 6 doublet (not "duplet") microtubules in F are in a linear array and thus have a non-random relationship to each other. I would guess that they represent 6 doublets that are linked laterally as if they started out as part of a set of 9 outer doublets that failed to

© 2023. Published by The Company of Biologists under the terms of the Creative Commons Attribution License (https://creativecommons.org/licenses/by/4.0/).

remain together during attempted assembly. In addition, because of their close relationship to each other, my guess is that the 2 singlet microtubules in 6F did not just happen to form near each other, but likely represent a central pair that failed to become incorporated into an intact axoneme. The authors might not agree with my interpretation of these images, but I think my interpretations are within the realm of possibilities and at least should be presented, along with reasons for discounting the idea.
We agree with the reviewer that there is a possibility that the 6 doublets shown in Figure 6F are linked, especially as there is no evidence that the absence of RSP9 would lead to incorrect linking of the microtubule doublets. Indeed, the electron dense shapes observed if Figure 6F may be similar to what can be seen in WT (Figure 6B-D). The current working hypothesis based on numerous observations by David Ferguson is that even in wildtype, axoneme formation is inefficient. This has been a major problem when examining the mutant parasites. Normal versus abnormal axonemes were counted in WT and mutant parasites, but normal axonemes were exceedingly rare in the mutant. We have now added a sentence with this hypothesis and amended the legend of Fig. 6F (line 341).

The word “duplet” has been replaced by “doublet”.

Along these lines, it would be helpful if the authors reminded the reader about current models of axoneme assembly and which step might require RSP9. I expect it has been shown the Plasmodium spp? basal body has triplet microtubules, and that the A and B tubules of each triplet provide that template for assembly of the outer doublet microtubules. For example, based on these results, my prediction would be that the doublets are nucleated in the mutant, that they form side-to-side associations as they extend, but that they fail to stay together as a 9-part unit because RSP9 provides an additional mechanism for connecting them? Thinking along these lines also brings to mind the question of whether or not dynein-driven microtubule sliding might force the doublets apart? Although not at all a requirement for this manuscript, in future experiments it would be interesting to learn whether intact axonemes would form in RSP9 mutants if they also lacked a dynein arm essential for motility?
We thank the reviewer for these insightful thoughts. We have now added a paragraph about radial spoke assembly in C. reinhardtii in the introduction (line 61).

In Plasmodium, the basal body consists of 9 single A-tubules and not triplets (reviewed by Sinden et al., Curr Opin Microbiol. 2010; 13:491–500, doi: 10.1016/j.mib.2010.05.016) and Francia et al., Cilia 201; 5:3, doi: 10.1186/s13630-016-0025-5). At the start of exflagellation, the centre of the basal body can be observed by electron microscopy only as an electron dense mass with singlet microtubules at the periphery (Sinden et al., Proc. R. Soc. Lond. B Biol. Sci. 1976, 193(1110):55-76, doi: 10.1098/rspb.1976.0031: Figures 18 and 19). The actual mechanism of axoneme assembly on the basal body is currently unknown.

Reviewer 2 Advance Summary and Potential Significance to Field:
Reviewer 2 Comments for the Author:

•The only major concern I have relates to conclusions about protein ‘requirement’ in T. brucei. For example, in the Abstract, lines 29-30: the statement that RSP9 orthologs are not necessary for axoneme assembly should be adjusted. The extent of knockdown (~40% in one case and ~70% in another) means that a substantial amount of mRNA remains present, and this level of expression could accommodate axoneme assembly. The authors are good at addressing this in the main text, e.g. line 205, but the statement in the abstract does not express this important caveat regarding presence of axonemes in RSP9/9L knockdowns.
Results in Leishmania, with complete KO, supports the general view, but care should still be taken in making the claim for T. brucei. A similar concern/caveat holds for lines 294-296. The statement says RSP9, 9L are "'both' necessary for RS head assembly...". Data indicate they are 'each' necessary in Leishmania, RSP9L is required in T. brucei, and the result is equivocal for RSP9 in T. brucei because no clear defect was observed, yet knockown efficacy was marginal. The statement needs to be re-worded accordingly.
Given the limited space in the abstract we cannot really clarify this - we argue that the consistent behaviour, albeit with the limitation of imperfect knockdown, of Lm and Tb support our overall conclusion and make this abstract text appropriate.

© 2023. Published by The Company of Biologists under the terms of the Creative Commons Attribution License (https://creativecommons.org/licenses/by/4.0/).

To take also into account a comment from reviewer 1, the last paragraph of the introduction has been modified to “Using gene deletion in L. mexicana, we show both RSP9 and RSP9-like are necessary for RS head assembly and normal cell motility but are not needed for cell survival. Similar results were obtained by RNAi knockdown in T. brucei.” (line 137).

To also answer a comment from reviewer 3, we added a sentence in the discussion “Due to a limited knockdown of T. brucei RSP9 and RSP9L expression in the RNAi mutants, we based our conclusions mainly on the knockout data from L.mexicana and use the T. brucei results as confirmation of the phenotype. ” (line 414).

Line 58: Grossman-Haham seems appropriate to also cite here.
This reference has been added.

Lines 65-66: It should be noted that motility is also required for progression through the insect vector and pathogenesis in the mammalian host (rotureau 2014b; shimogawa 2018 that are cited here). To a general audience, that is likely not clear from simply saying "life cycle".
This sentence has been modified to “In T. brucei all life cycle stages have a motile flagellum, which is essential for motility, but also for cell morphogenesis and progression through the insect vector, as well as for pathogenesis in the mammalian host. ” (line 75).

Line 104: is it accurate to say plasmodium display "canonical" 9+2 axonemes? In the EM images presented (Fig 6 and S3) it isn't clear if they have canonical spokes. Even the fuzzy electron dense material seen between outer doublets and central pair appears to extend more from the dyneins, rather than the doublet. Having only 3 of 23 or more canonical radial spoke proteins, it is hard to imagine plasmodium having canonical spokes. it's perhaps a nuance, but would be worth adjusting this comment, and this could be very informative and
enlightening for the community, as a solid description of the wt axoneme would be of interest.
We would like to clarify that when we speak about “canonical” 9 + 2 axonemes, we refer the arrangement of the microtubules rather than the whole structure including radial spokes and dynein arms etc. We have now added “microtubule” before architecture to make this clear (line 122).

Line 170: should read "...and that TbRSP9 and PbRSP9 PREDICTED structures
are very similar to..."
The sentence has been changed to “TbRSP9 and PbRSP9 predicted structures are very similar to C. reinhardtii ” (line 203).

Methods 5.10:please include the reference for motility sedimentation assay in the methods section. The section itself is quite brief, understandably if this is a standard assay, but a reference for a more complete description should be provided.
The sedimentation assay has been more described and the reference for the test has been included. It now reads “For motility analysis in T. brucei, a sedimentation assay was realised as previously described by (Ralston et al., 2006). 5x106 uninduced and 6 day induced trypanosomes were incubated at 27°C in 1 mL medium in spectro-photometric cuvettes and optical density was measured every 2 hours compared to control in which cells were resuspended.” (line 697).

Line 571: For ookinete formation, it would be helpful to see representative images of the distinguishing features used to quantitate ookinetes.
The staining with fluorescently-labelled anti-Pbs28 antibody can be used on macrogametocytes, zygotes, retorts and ookinetes. We have used the same criteria as shown in Figure 2b of following paper: https://doi.org/10.1186/s13071-016-1932-4.

•RESULTS, subheading 3.5.
•The heading should be adjusted to reflect the finding that spoke heads
are affected. The heading simply states ...not essential for axoneme structure...
We have rephrased this title to include the RS head result. “RSP9 is essential for RS head formation which disrupts axoneme formation in Plasmodium but not Trypanosoma and Leishmania” (line 316).

•The mixed cross using defined female-defective or male-defective plasmodium mutants is clever and informative

© 2023. Published by The Company of Biologists under the terms of the Creative Commons Attribution License (https://creativecommons.org/licenses/by/4.0/).

We thank the reviewer for this encouraging comment.

Fig. 1: It would be informative to include the alpha fold prediction for the Chlamydomonas protein, to see how this compares to the experimentally determined structure.
The structure in Figure 1C is the Cr Alphafold-predicted structure - the acidic loop is likely unstructured (low pLDDT) and was not resolved in the cryo EM structure. All structures are shown aligned with the experimentally-determined structure, shown as a gray 'ghost'. This wasn't explicitly stated in the figure legend, and we have clarified this.

Fig 4: The legend appears to have a typo, with panel F labeled as panel 'b. Fig 4, panel F is problematic: There is not sufficient clarity to discern differences between the wt and rsp9-KO. one panel of the KO looks unlike the other three panels, but the other three panels look, for most purposes, to be similar. the authors need to provide a) examples of whole fields of samples and some quantitative analysis, to evaluate how representative the stated defect is; and b) a descriptive panel in which the relative features that are important are clearly labeled. The result is a central finding of the work and needs some more demonstrated support.
We have corrected the wrong labelling of the figure legend 4F. Reviewer 1 raised a similar point. We have addressed this above.

Reviewer 3
Summary of the paper:
The authors investigated heterogeneity in radial spoke proteins (RSPs) across several species and then performed an investigation into the role of the orthologs of RSP9 in Plasmodium and trypanosomatids. They verified RSP9 ortholog localization to flagella and discovered that loss of the RSP9 ortholog had differential effects on flagella depending on the organism. In T. brucei and L. Mexicana flagella formed however swimming was impaired. In P. berghei flagellar assembly was disabled. The authors conclude that the different requirements for RSP9 are due to different selection pressures related to the mechanism of flagellar assembly.
As written the manuscript does not meet the Journal standard for making a significant and novel contribution to our understanding of cell biology of broad interest. However, I am optimistic that a revised manuscript could meet the standard. I have provided details below, but in general, the motivation and significance of the research are under-developed and thebioinformatic and experimental studies are insufficiently integrated. In addition, there are technical concerns with the data presented that I hope can be easily addressed.

Motivation and significance:
• The motivation listed in the abstract is "flagella are important for motility". Because you are studying parasites that require flagella for their life cycle, and investigating difference, a more extensive motivation could be presented.
Flagella are indeed vital for all of the parasites we analyse here, and have added that to the first line of the abstract. We have also clarified in the abstract that there are striking differences in the biology of trypanosomatid and Plasmodium flagella which may correlate with differences in structure.

• To more clearly communicate the value of the work you have done, define the unanswered question that you answer in this study (include it in the abstract, introduction and answer it in the discussion). In the Intro you write: "it is not known precisely why RSs are important..." This study does not answer that question.
We agree that the abstract was over-focused on RSP9 when we carried out a wide bioinformatic survey of RSPs to identified RSP9 as an uncharacterised protein of interest. This is specifically related to the question of flagellum adaptations in a long-lived vs transient, canonically vs non-canonically assembled and constitutively present vs. one life cycle stage only flagellum. We cannot answer this larger question in full, but address these points in the discussion.

Establish a motivation for examining functional conservation of RSP9 after identifying all RS in so many species.
RSP9 was selected due to the surprising presence of two divergent orthologs in trypanosomatids, its conservation in Plasmodium and because it hasn't been the subject of analysis in either group of parasites. We hope that this would have been clear from the text, however this was evidently not the case and we have made a variety of changes to clarify this selection process.

© 2023. Published by The Company of Biologists under the terms of the Creative Commons Attribution License (https://creativecommons.org/licenses/by/4.0/).

Integration of bioinformatic and experimental analysis:
In the current draft, the bioinformatic analysis competes with the analysis of RSP9 instead of motivating or complimenting it. Here are a few specific points, but the integration may require more than just addressing these.
The bioinformatic analysis is a vital foundation for the later work. We could, for example, move Figure 1A to supplementary material to deprioritise its presentation – however we think that 'hides' the useful presentation of overall conservation of RSPs across eukaryotes.
Regarding the text we have made a variety of changes to better explain the overall workflow.

• Why is a comprehensive analysis of diverse organisms useful to identify the orthologs in 4 species to the RSPs in Chlamydomonas?
This is necessary as a control – detection of orthologs in non-flagellate species indicates members of that protein have non-flagellar functions while failure to detect orthologs in flagellate species indicates rapid sequence divergence or lineage-specific components. We did not carry out a quantitative analysis of this, but have added text to clarify the importance of analysing many species.

• Results from the bioinformatic analysis are detailed both in the second to last paragraph of the introduction as well as the first 3 paragraphs of the Results section
This was intended as a results summary at the end of the introduction. We have shortened this text to avoid the perception of this as duplicated results.

• 4 of the 5 supplemental figures are not about RSP9
We do not agree with the reviewer here: figures S1, S2 and S5 all involve RSP9. Figure S1 and S2 do show many other RSPs and act as more foundational analysis, they have therefore been added as supplemental rather than main figures.
The analysis of the RSP11-like candidate in Plasmodium could be removed (S3, S4) as it does not affect the overall conclusions, however this is useful negative data for future research into RSPs and we would believe that negative results, if they are connected to the main data, should be made public. Moreover, RSP11 links well with the comprehensive bioinformatic gather we carried out.

One possible strategy for you to consider:

There are multiple ways that one could more clearly communicate the motivation and significance of the study and to integrate the different approaches. Here is one possibility that occurred to me.
• All motile cilia and flagella have RS, but they are not the same in all species. In species examined to date there is variability between the component proteins

• Loss/mutation of RSPs causes defects in conventional model organisms suggesting mechanical/mechanosensitive function. It is possible that the RS has the potential to serves multiple functions within a single flagellum

• Unanswered question: How do RSPs contribute differently to flagellar assembly and function in parasite species that depend on flagella for life cycle?
• Use bioinformatics to identify an RSP that can be used to explore this conundrum
• Identify RSP9
This is essentially the structure and logic that we intended to convey, with the nuance that there is little point in re-analysing RSP4/6 or 3 in trypanosomatids, where these have been experimentally characterised previously, and that RSP9 stands out as interesting because of the duplication in trypanosomatids.
We have made a variety of changes, mostly some changes to paragraph structure/paragraph first sentences and have moved one subtitle to clarify this structure.

We agree that a very short paper could be written which addresses only these specific points as a purely comparative study. However as important pathogens, there is significant value to allow space for discussing the parasite lineage-specific implications – particularly for Apicomplexa.

© 2023. Published by The Company of Biologists under the terms of the Creative Commons Attribution License (https://creativecommons.org/licenses/by/4.0/).

• In line 319 (Discussion) the statement that "the overall structure of RSs, as visualized by electron microscopy, is very well conserved across all ciliated/flagellated organisms..." seems contradicted by visual differences in RS in Figure 5.

The overall structure (which we have changed to shape, as structure may imply molecular architecture) of radial spokes is very similar across eukaryotes. Figure 5 shows that differences between wild type Tb and Lm are minimal, and that most differences are in deletion/RNAi knockdown mutants. We do not see the contradiction in these statements.

Important issues with the data that need to be addressed:
• Two Plasmodium species are used in this study. It is unclear which is examined in some figures and why each was analyzed where it was. In figure 1B, C, & D, Plasmodium berghei is featured. In figure 2 there are images of Plasmodium falciparum. I think Figure 4 and 6 feature P. berghei because line 231 states that the knock-out lines are made in that organism, however this is not indicated anywhere else in the results, figure legend or figure.
We apologize that this point has not been clearly stated. All the main experimental work has been performed with Plasmodium berghei as this mouse strains is more readily amenable to genetic manipulation and not hazardous to work with mosquito stages. For Figure 2C, we have added a new header stating that this is P. berghei as done for all other panels of this figure. We have also modified the figure legend.

We have generated antibodies against P. falciparum RSP9 to be able to study this protein in P. falciparum and P. berghei, expecting crossreactivity to P. berghei RSP, which we eventually did not observe and thus used P. falciparum instead. We have now specified the species more clearly in figure legend 2 and in the main text.

• **The staining in Figure 2D is not convincing.** What is the intense central signal that is not similar to the alpha tubulin. Include data or a reference to support that the PfRSP9 antisera is specific for RSP9. Also include additional example images.

We agree with the reviewer that the intense staining of the gametocyte body is surprising. We hypothesise that this comes from RSP9 that is either a pool of RSP9 monomers or RSP9 already assembled in the 12S complex, but not yet transported to the flagella (see also line 61) and more present in abundance. The antibodies were raised against peptides and were tested in Western blots:

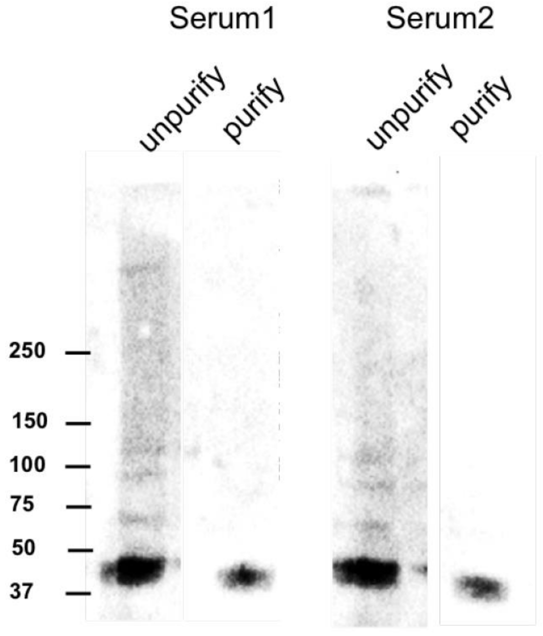

© 2023. Published by The Company of Biologists under the terms of the Creative Commons Attribution License (https://creativecommons.org/licenses/by/4.0/).

The Western blots show clearly bands that correspond to the expected 42 kDa of P. berghei RSP9 (unpurify refers to serum, purify to protein A-purified antibody). Additionally, we show the expected absence of staining in ring and schizont stages in figures 2E and F.

For the publication, we have chosen the best examples although we have more. We do not believe that displaying more images adds value to the manuscript, but are happy to show some more images here:

DAPI anti-RSP9 anti-tubulin Merge

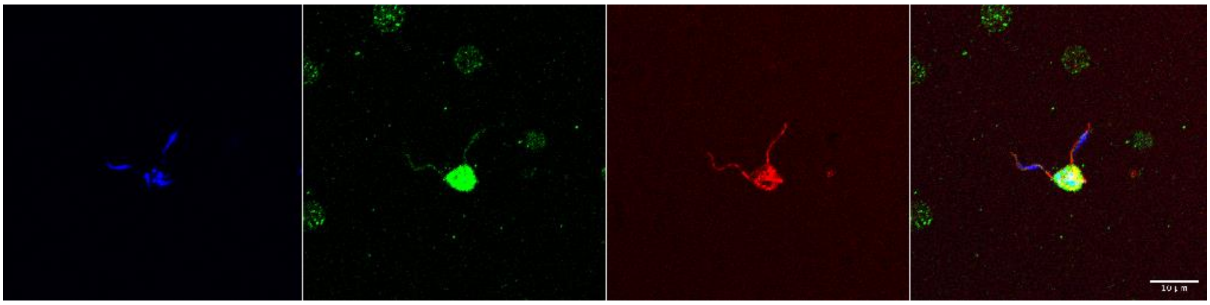

• **Figure 3 A and C** should include data from replicates with statistical analysis instead of representative plots.
We have modified the figure accordingly and show now statistical data from independent experiments.

• The conclusions drawn from the T. brucei knock downs (including lines 218 & 219 and the discussion) should be moderated to account for the partial knockdown. One cannot conclude that removal of the protein has no affect on assembly when the protein has not been completely removed.
We moderated all the conclusions from the T. brucei knockdown and agree with the reviewer that partial knockdown does not allow the conclusion that "removal of the protein has no effect on assembly when the protein has not been completely removed". We state this clearly in the results section "Although this represents only limited knockdown efficiency, the partial absence of structural proteins such as RSP9 or RSP9L could still have an effect of the axonemal stability. Analysis of these cell lines was done with this caveat in mind." (line 237). We added a sentence in the discussion "Due to a limited knockdown of T. brucei RSP9 and RSP9L expression in the RNAi mutants, we base our conclusions mainly on the knockout data from L. mexicana and use the T. brucei results as confirmation of the phenotype. " (line 414).

• The EM presented in Figure 6 is essential and should be expanded.
Indeed, we agree that Figure 6 is a key figure of this manuscript. However, we believe that we show representative images that are also described in the text. We believe that adding more images would make Figure 6 simply more convoluted and would not add much value to the message.

• Fig. 6E in the legend is described as a section showing the absence of axonemes.
How is this consistent with the IF shown in Figure 4 where the flagella are described as "either wrapped around the gametocyte body or protruding only partially"?
We are not claiming to show the absence of axoneme associated protein/ microtubules – in fact in the EM, we are trying to show the exact opposite in that numerous doublet and even central microtubules are present – it is just that there are various degrees of disorganisation. 9+2 (normal axonemes) were exceedingly rare in the RSP9 mutant and there is still some formation and partially association of doublet microtubules and potentially the double central microtubules (Fig 6F). Certain of these microtubule combinations can partially "exflagellate" with portions of cytoplasm containing disorganise doublet microtubules (Fig 6H and I). These observations are consistent accord with the IF images shown in Fig 4 (see also below).

• Have all of the sections been assessed?

© 2023. Published by The Company of Biologists under the terms of the Creative Commons Attribution License (https://creativecommons.org/licenses/by/4.0/).

Numerous and multiple (but not serial) sections of both a number of both WT and mutant microgametocytes were examined. While normal 9+2 axonemes were seen in WT parasites exceedingly few were observed in a similar number of sections examined in the mutant.

• Can you make more connections between how the EM explains what you see by IF?
The reviewer has a very valid point: Whilst in the IFA of the mutant parasites, we can observe either no protruding flagella or flagella protruding partially but without associated nuclei, the EM sections show disruption of the canonical 9 + 2 microtubule structure. We can only hypothesize that disruption of the microtubular structure leads to instable structures that are incapable of recruiting or tethering a nucleus. We have now added a sentence to formulate this hypothesis (line 354).

• Are basal bodies present or disrupted?
Our EM images are mainly show cross sections of the axoneme where we expected to see the effect of the introduced knock-out. RSP9 being predicted to be a radial spoke head component, we do not believe that the basal body is disrupted. However, we have however not directly addressed this question due to the challenges of identifying basal bodies with confidence at all in gametocytes.

• I really like Figure 5, by the way. It is presented and described clearly and the differences are interesting!
We thank the reviewer for this constructive and encouraging comment.

Minor Points:
• Include catalog numbers in the methods section – especially for antibodies.
We have now added manufacturer names or references for the antibodies used.

• In every figure legend indicate the organisms that have been used. For example, in Figure 3, T. brucei and L. Mexicana are only identified in the figure title and should be included to indicate that A, B and C are T. brucei and D-G are L. Mexicana.
We have added species names to the figure legend panel descriptions. Note, that we only identified this information as missing for Figure 3 and 5. In those, the cell line names, including two letter species identification, were already clearly stated.

• Line 770: “(E-!)” should be “(E-I)”
This has been corrected.

Second decision letter

MS ID#: JOCES/2022/260655

MS TITLE: Radial spoke protein 9 is necessary for axoneme assembly in *Plasmodium* but not in trypanosomatid parasites

AUTHORS: Chandra Ramakrishnan, Cecile Fort, Sara Rute Marques, David JP Ferguson, Marion Gransagne, Jake Baum, Soraya Chaouch, Elisabeth Mouray, Linda Kohl, Richard John Wheeler, and Robert E Sinden
ARTICLE TYPE: Research Article

We have now reached a decision on the above manuscript.

To see the reviewers' reports and a copy of this decision letter, please go to: https://submit-jcs.biologists.org and click on the 'Manuscripts with Decisions' queue in the Author Area. (Corresponding author only has access to reviews.)

As you will see, the reviewers gave favourable reports but raised some critical points that will require amendments to your manuscript. I hope that you will be able to carry these out because I would like to be able to accept your paper, depending on further comments from reviewers.

© 2023. Published by The Company of Biologists under the terms of the Creative Commons Attribution License (https://creativecommons.org/licenses/by/4.0/).

Please ensure that you clearly highlight all changes made in the revised manuscript. Please avoid using 'Tracked changes' in Word files as these are lost in PDF conversion.

I should be grateful if you would also provide a point-by-point response detailing how you have dealt with the points raised by the reviewers in the 'Response to Reviewers' box. Please attend to all of the reviewers' comments. If you do not agree with any of their criticisms or suggestions please explain clearly why this is so.

<u>Reviewer 1</u>

Advance summary and potential significance to field

As described in my first review.

Comments for the author

The authors have adequately and appropriately addressed my original comments and concerns.
One comment about grammar:
I think that the subjunctive mood is being used in the following sentence in the abstract "We asked if there are radial spoke adaptations associated with parasite lineage-specific properties in apicomplexans and trypanosomatids." Thus, I think the sentence should be "We asked if there were radial spokes . . .. ."
ChatGPT agreed with me.
Of course on this minor point, I am happy to let the authors decide. (And, ChatGPT be damned!)

<u>Reviewer 2</u>

Advance summary and potential significance to field

Analysis of flagellum structure and function in Plasmodium is a much needed effort and the work will inform biology of this deadly pathogen, as well as advance understanding of flagellum assembly mechanisms.

Comments for the author

The revised paper is improved through the authors’ work and revisions. There are however, important concerns that remain unaddressed.
1) My prior concern regarding clarity in the Abstract remains unaddressed, and I still find the Abstract writing unacceptable. Regarding the authors'
suggestion that they do not have room to clarify, I expect the authors are clever and capable enough to prepare an abstract that does not misrepresent data/conclusions while staying within the prescribed word limits. Likewise, the statement regarding line 137 that
"...similar results were obtained by RNAi knockdown in T. brucei”, is simply not accurate and can easily be adjusted to be accurate.
2) Regarding Fig. 4F. I do not see where my concern from the original review is addressed, and I remain unconvinced by the data shown of the authors' claim (line 299-302) that this Fig 4F shows differences claimed for WT vs deltaRSP9 e.g. "WT flagella were clearly stained with both anti-α-tubulin II antibody and DAPI revealing a wavy structure typical of motile gametes. While Δrsp9 have clearly distinguishable nuclei that reside in the main male gametocyte body, their flagella remain either wrapped around the gametocyte body or protrude only partially without nuclei being associated (Fig.
4F)." To the contrary, it seems that the above statements about WT could apply to the mutant and vice versa. Some nuclei within the WT sample seem to be within the cell body and not clearly associated with flagella, while I would be hard-pressed to say that mutant flagella protrude less than WT. See my original comments for recommendations to address this concern.

© 2023. Published by The Company of Biologists under the terms of the Creative Commons Attribution License (https://creativecommons.org/licenses/by/4.0/).

Reviewer 3

Advance summary and potential significance to field

The revised manuscript is significantly better than the original submission. The modifications made by the authors now communicate that this study addresses a question that will be of interest to the readers of Journal of Cell Science. The changes to the text now better integrate the search for orthologues and the experimental investigations. I list a few remaining thoughts below, which if addressed, would qualify the manuscript for publication in JoCS.

1) Please indicate in Figure 1A which species are non-flagellate.
In the response to reviewers the authors contend that the value of the extensive analysis is that a comparison can be made between species with and without flagella. I understand their argument, however, the current version of the figure assumes the readers will know which species are non-flagellate.

2) It can be helpful to other people in the field to see the validation of tools, such as antibodies, so I recommend including at least the western blots in the supplement. In the version of the response to reviewers I received, the images included by the authors were absent. Perhaps the editor can see these images and evaluate the western blots and additional images presented.

Comments for the author

In Figure 1 the colors representing Rsp2, 3, and 16 are similar. This is a new comment that I didn't raise in my initial review, however, I think it would be valuable to make Rsp3 stand out more.
Small Corrections:
1) I think the second pair should have an “L”:
Line 199/200 TbRSP9/LmRSP9 and TbRSP9/LmRSP9
2)remove the word "that"
Line 210 …we sought to determine whether that T. brucei

Second revision

Author response to reviewers' comments

We thank you for encouraging us to submit a newly revised version of manuscript JOCES/2022/260655. We also appreciate that the reviewers were attentive to the details and we hope we have addressed all points adequately now. We have addressed all the newly raised issues point by point and have also added text to the manuscript accordingly. The major changes are:
- Changed colours in Figure 1B.
- Addition of a supplemental figures containing a Western blot validating the anti-PFRSP9 anti-sera.
- In Figure 4F, there is now one additional image for WT male gametocytes to further illustrate the difference to the mutant.
- Added text for clarity.
- Removed text that has been replaced (crossed out).

All new changes are listed in our rebuttal below and in the main text; these changes in the main text are highlighted in light blue while the previous changes from the last revision remain highlighted in yellow.

We thank you and the reviewers for putting in a tremendous effort and assisting us in improving this manuscript. We also thank you for considering this new version for publication in the Journal of Cell Sciences.

© 2023. Published by The Company of Biologists under the terms of the Creative Commons Attribution License (https://creativecommons.org/licenses/by/4.0/).

Reviewer 1 Advance Summary and Potential Significance to Field:
As described in my first review.

Reviewer 1: Comments for the Author
The authors have adequately and appropriately addressed my original comments and concerns.

One comment about grammar:
I think that the subjunctive mood is being used in the following sentence in the abstract "We asked if there are radial spoke adaptations associated with parasite lineage-specific properties in apicomplexans and trypanosomatids." Thus, I think the sentence should be "We asked if there were radial spokes . . .. ."
ChatGPT agreed with me.

Of course on this minor point, I am happy to let the authors decide. (And, ChatGPT be damned!)
We have corrected this in the main text (line 28).

Reviewer 2: Advance Summary and Potential Significance to Field
Analysis of flagellum structure and function in Plasmodium is a much needed effort and the work will inform biology of this deadly pathogen, as well as advance understanding of flagellum assembly mechanisms.

Reviewer 2: Comments for the Author
The revised paper is improved through the authors' work and revisions. There are, however, important concerns that remain unaddressed.

1) My prior concern regarding clarity in the Abstract remains unaddressed, and I still find the Abstract writing unacceptable. Regarding the authors' suggestion that they do not have room to clarify, I expect the authors are clever and capable enough to prepare an abstract that does not misrepresent data/conclusions while staying within the prescribed word limits.
We thank the reviewer for his 'confidence' in our capabilities. We have amended the text in a way that data is not misrepresented and hope that the reviewer agrees with us. Specifically, we have replaced the sentence "Using Trypanosoma brucei and Leishmania mexicana, we demonstrate that trypanosomatids have an extensive RSP complement including two divergent RSP9 orthologs, both necessary for flagellar beating and swimming, but not axoneme assembly." with "Trypanosoma brucei and Leishmania mexicana, have an extensive RSP complement including two divergent RSP9 orthologs, necessary for flagellar beating and swimming. Detailed structural analysis showed that neither ortholog is needed for axoneme assembly in Leishmania."

Likewise, the statement regarding line 137 that"...similar results were obtained by RNAi knockdown in T. brucei", is simply not accurate and can easily be adjusted to be accurate.
We understand the reviewer's point and have replaced the sentence with a more elaborate explanation (line 143 onwards). However, we would like to add that we - according the reviewer's comment after the original submission as well as a point raised by reviewer 3 before – caution the readers (line 243 onwards) where we have already added a sentence in the first revision to clarify this point ("Although this represents only limited knockdown efficiency…"). We also mention this further (line 334 and 417 onwards: "Bearing in mind the limited RSP9 RNAi knockdown in T. brucei,…" and "Due to a limited knockdown of T. brucei RSP9 and RSP9L expression in the RNAi mutants…". Reviewer 3 also commented on the first version of the discussion that the limited knockdown in T. brucei has to be taken into account for which we had added a sentence in the first revised version of the manuscript (line 417 onwards).

2)Regarding Fig. 4F. I do not see where my concern from the original review is addressed, and I remain unconvinced by the data shown of the authors' claim (line299-302) that this Fig 4F shows differences claimed for WT vs deltaRSP9,e.g. "WT flagella were clearly stained with both anti-a-tubulin II antibody and DAPI revealing a wavy structure typical of motile gametes. While ?rsp9 have clearly distinguishable nuclei that reside in the main male gametocyte body, their flagella remain either wrapped around the gametocyte body or protrude only partially without nuclei being associated (Fig. 4F)." To the contrary, it seems that the above statements about WT could apply to the mutant and vice versa. Some nuclei within the WT sample seem to be within the cell body and

© 2023. Published by The Company of Biologists under the terms of the Creative Commons Attribution License (https://creativecommons.org/licenses/by/4.0/).

not clearly associated with flagella, while I would be hard-pressed to say that mutant flagella protrude less than WT. See my original comments for recommendations to address this concern.
We understand the reviewer's concern. Indeed, we should have outlined better that nuclei still residing or remaining in the gametocyte body can also happen in WT as shown in Figure 4F formerly on the very right (the figure is representative), but that nuclei associated with the flagella have never observed in the mutant. We however agree that maybe the claim may be too strong with no numbers to back up, so we will omit statements about no observed associated nuclei in the mutant. We also have now re-arranged the figure and added a third image for WT so that the figure with the nuclei in the gametocyte body does not carry the same weight.

We have also gone back to your previous comment: "The images should be larger and the text needs to do a better job of describing and labeling the flagella for a reader not familiar with viewing Plasmodium spp? exflagellating male gametoctytes."
We are not able to have larger images, the resolution of these images does not allow magnification. We agree with the reviewer that the images ideally should have been captured at higher resolution, but this does not affect our core conclusions. We have however re-modified the text that now reads: "At 15 min post-activation, WT flagella were clearly stained with both anti-a-tubulin II antibody and DAPI revealing a wavy structure typical of motile gametes. Nuclei are mostly associated to flagella although in some cases, nuclei can be observed residing or remaining in the main male gametocyte body, consistent with the prior observation that ~50% of free gametes are anucleate in wildtype parasites (Sinden 1975). While ?rsp9 show extensive staining with anti-a-tubulin II antibody and have clearly distinguishable nuclei their flagella seem to protrude only partially. Most importantly, free male gametes have never been observed (Fig. 4F). Mitosis therefore appears to be completed normally, while subsequent axoneme motility appears to be abolished." We believe that with these amendments, the message is clearer and thank the reviewer for his/her suggestions.

Reviewer : Advance Summary and Potential Significance to Field

Reviewer 3 Advance Summary and Potential Significance to Field:
The revised manuscript is significantly better than the original submission. The modifications made by the authors now communicate that this study addresses a question that will be of interest to the readers of Journal of Cell Science. The changes to the text now better integrate the search for orthologues and the experimental investigations. I list a few remaining thoughts below, which if addressed, would qualify the manuscript for publication in JoCS.
We thank the reviewer for the encouraging feedback.

1) Please indicate in Figure 1A which species are non-flagellate. In the response to reviewers the authors contend that the value of the extensive analysis is that a comparison can be made between species with and without flagella. I understand their argument, however, the current version of the figure assumes the readers will know which species are non-flagellate.
These species were selected based on availability of genomic data and eukaryotic diversity. While it is generally known whether they are ciliate, there are a few where a flagellate life stage is not known but it seems likely from the genome that there is one. We have not added an indication of non-flagellate species to the figure as this may be taken as evidence that some of these species are aflagellate when in some cases that is not (yet) unambiguously known.
2) It can be helpful to other people in the field to see the validation of tools, such as antibodies, so I recommend including at least the Western blots in the supplement. In the version of the response to reviewers I received, the images included by the authors were absent. Perhaps the editor can see these images and evaluate the western blots and additional images presented.
We have checked our previous rebuttal letter and an explanatory Western blot had been included. Nonetheless, we have now added a supplemental figure (Fig. S6) to the manuscript where the anti-sera against Plasmodium falciparum RSP9 have been validated. The methodology is now also described in the Materials and Methods section (line 691 onwards). We have now also included two more images of WT male gametocytes to Fig. 4F to better illustrate the knock-out phenotype in contrast to WT.

© 2023. Published by The Company of Biologists under the terms of the Creative Commons Attribution License (https://creativecommons.org/licenses/by/4.0/).

Reviewer 3: Comments for the Author

In Figure 1 the colors representing Rsp2, 3, and 16 are similar. This is a new comment that I didn't raise in my initial review, however, I think it would be valuable to make Rsp3 stand out more.

We have now changed the colours so that RSP3 is more visible.

Small Corrections:

1) I think the second pair should have an “L”: Line 199/200 TbRSP9/LmRSP9 and TbRSP9/LmRSP9

We thank the reviewer for having spotted this mistake. We have corrected this now (new line 206).

2) remove the word "that": Line 210 …we sought to determine whether that T. brucei

This is now corrected (new line 216).

Third decision letter

MS ID#: JOCES/2022/260655

MS TITLE: Radial spoke protein 9 is necessary for axoneme assembly in *Plasmodium* but not in trypanosomatid parasites

AUTHORS: Chandra Ramakrishnan, Cecile Fort, Sara Rute Marques, David JP Ferguson, Marion Gransagne, Jake Baum, Soraya Chaouch, Elisabeth Mouray, Linda Kohl, Richard John Wheeler, and Robert E Sinden

ARTICLE TYPE: Research Article

I am happy to tell you that your manuscript has been accepted for publication in Journal of Cell Science, pending standard ethics checks.

© 2023. Published by The Company of Biologists under the terms of the Creative Commons Attribution License (https://creativecommons.org/licenses/by/4.0/).
